# Synthesis of sodium polyhydrides at high pressures

**DOI:** 10.1038/ncomms12267

**Published:** 2016-07-28

**Authors:** Viktor V. Struzhkin, Duck Young Kim, Elissaios Stavrou, Takaki Muramatsu, Ho-kwang Mao, Chris J. Pickard, Richard J. Needs, Vitali B. Prakapenka, Alexander F. Goncharov

**Affiliations:** 1 Geophysical Laboratory, Carnegie Institution of Washington, 5251 Broad Branch Road NW, Washington, District of Columbia 20015, USA; 2 Center for High Pressure Science and Technology Advanced Research, Shanghai 201203, China; 3 Lawrence Livermore National Laboratory, Material Sciences Division, 7000 East Avenue, L-350, Livermore, CA 94550-9698, USA; 4 Department of Physics and Astronomy, University College London, Gower Street, London WC1E 6BT, UK; 5 Department of Materials Science & Metallurgy, University of Cambridge, 27 Charles Babbage Road, Cambridge CB3 0FS, UK; 6 Theory of Condensed Matter Group, Cavendish Laboratory, J J Thomson Avenue, Cambridge CB3 0HE, UK; 7 Center for Advanced Radiation Sources, The University of Chicago, Chicago, Illinois 60637, USA; 8 Key Laboratory of Materials Physics, Institute of Solid State Physics, Chinese Academy of Sciences, Hefei, Anhui 230031, China

## Abstract

The only known compound of sodium and hydrogen is archetypal ionic NaH. Application of high pressure is known to promote states with higher atomic coordination, but extensive searches for polyhydrides with unusual stoichiometry have had only limited success in spite of several theoretical predictions. Here we report the first observation of the formation of polyhydrides of Na (NaH_3_ and NaH_7_) above 40 GPa and 2,000 K. We combine synchrotron X-ray diffraction and Raman spectroscopy in a laser-heated diamond anvil cell and theoretical random structure searching, which both agree on the stable structures and compositions. Our results support the formation of multicenter bonding in a material with unusual stoichiometry. These results are applicable to the design of new energetic solids and high-temperature superconductors based on hydrogen-rich materials.

## Abstract (web summary)

The only known compound of sodium and hydrogen is ionic NaH, but theory predicts the existence of polyhydrides at high pressure. Here, the authors report observations of the formation of polyhydrides above 40 GPa and 2000 K, supporting the idea of multicentre bonding in a material with unusual stoichiometry.

## Graphical abstract

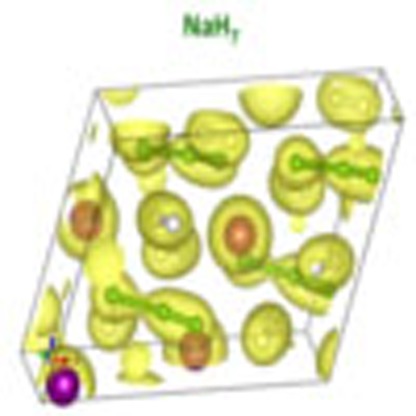

Dense hydrogen is of central interest in many disciplines, especially in high-pressure science. It is expected to possess unusual properties such as high energy density^1^, high-temperature superconductivity and superfluidity^2^. Unusual high-pressure properties may be sustained at ambient conditions, if a predicted metastable metallic phase of hydrogen^3^ could exist at ambient pressure. This phase would have unusual anisotropic structure, consisting of weakly interacting chains of hydrogen atoms with interatomic distances ∼1.06 Å (ref. 3). Looking for another route to force hydrogen into a metallic state, Ashcroft^4^ proposed that such conducting states could be realized in hydrogen-rich alloys, where hydrogen is in a ‘pre-compressed' or otherwise altered electronic states induced by the host material (‘dopant') in such a way, that the electronic bands of hydrogen and the host element(s) overlap at the Fermi level. For example, polyhydrides LiH_2_ and LiH_6_ (refs 5, 6) were predicted to have stable semi-metallic (LiH_2_) and metallic (LiH_6_) phases above 100 GPa, which is nearly four times lower than the calculated metallization pressure of pure hydrogen^7^. It should be noted that in lithium polyhydrides the metallization does not occur due to ‘precompression' of hydrogen, but rather due to ‘doping' by electropositive elements^5^,^6^. In extension of these ideas, recent theoretical analysis of MH_n_ (M=Li, Na, K, Rb, Cs, Sr) compounds with variable hydrogen composition resulted in prediction of stable polyhydrides of alkali and alkaline earth metals^5^,^6^,^8^,^9^,^10^,^11^,^12^,^13^.

The compounds with more than two hydrogen atoms per alkali atom are expected to become stable at pressures as low as 25 GPa in the case of Na and above 100 GPa in the case of Li. Hydrogen-rich polyhydride phases are also predicted at high pressures for alkaline earth metals^10^,^14^,^15^. Such hydrogen-rich polyhydride materials are stabilized by compression, and many of them are expected to become metallic and superconducting at lower pressures than pure hydrogen. For example, high critical superconducting temperatures (*T*_c_∼235 K) are predicted for polyhydrides of Ca (ref. 14). Moreover, the recent discovery of superconductivity in hydrogen sulfide at a record *T*_c_∼203 K at high pressure (150 GPa) has confirmed the great potential of dense hydride materials as high-temperature superconductors^16^. Thus, the recently predicted polyhydride compounds may pave the route to alter the electronic structure in a way that facilitates the creation of metallic superconducting materials with record high critical superconducting temperatures^4^,^17^.

One of the salient features predicted to form in polyhydrides of heavier alkali metals is a motif of linear H_3_^−^ ions (such ions are predicted to form in RbH_5_ (ref. 18) and CsH_3_ (ref. 11)). H_3_^−^ ions are known to exist in a linear configuration, while H_3_^+^ ions form a triangular-shaped unit^19^,^20^. Notably, triangular H_3_^+^ ions were predicted to be stable in the H_5_Cl compound^21^ at high pressures up to 300 GPa. The symmetric linear H_3_^−^ ions^22^ were discussed as transition states in hydrogen exchange processes of metal complexes^23^. On the experimental side, H_3_^−^ and D_3_^−^ ions were observed in discharge plasmas only recently^24^. It should be noted here, that ambient pressure metastable metallic hydrogen phases predicted in 1972 by Browman and Kagan^3^ are composed of one-dimensional hydrogen chains, which are similar to the chains of H_3_^−^ ions predicted theoretically in RbH_5_ (ref. 11) and CsH_3_ (ref. 17). The simplest model of strong correlations in a linear chain of hydrogen atoms^25^,^26^ is also based on similar equidistant chain motifs. Despite a wealth of theoretically predicted high pressure polyhydride structures, none of the predictions has been confirmed until now, except possible Li polyhidride phases. The polyhydrides of Li were reported recently, based on the measurements of the infrared absorption spectra of LiH by Pepin *et al*^27^. New absorption bands observed in their work above 130 GPa are consistent with the calculated infrared modes in LiH_6_ and LiH_2_. The new polyhydrides of Li have been produced by compression of LiH in a rhenium gasket without any pressure medium. Both compounds remain optically transparent to 215 GPa, which is at odds with calculations^28^. The authors did not attempt to characterize their samples by x-ray diffraction method and Raman spectroscopy, which makes it difficult to estimate if they had significant amounts of the reacted materials in the high pressure samples.

Here we report the synthesis of Na polyhydrides at pressure of ∼30 GPa in laser-heated diamond anvil cell (DAC) experiments at temperatures above 2,000 K. We were guided by *ab-initio* theoretical search, which yielded a number of stable NaH_*x*_ (*x*=1.5–13) materials (Fig. 1) more favourable than those predicted previously^8^. In agreement with these predictions, we identified the NaH_3_ solid using *in sit*u synchrotron X-ray diffraction (XRD) measurements. Moreover, both XRD and Raman spectroscopy revealed the presence of the NaH_7_ phase, which has a characteristic Raman band at 3,200 cm^−1^, suggesting the formation of H_3_^−^ ions. Our results therefore provide the first verification of the existence of polyhydrides of alkali metals with heterogeneous (multicenter) chemical bonding and prospects for lower pressure metallization.

## Results

### X-ray diffraction experiments and Raman measurements

Several experiments were performed with Li and Na samples up to 70 GPa at room temperature. In these runs, only the formation of LiH and NaH was detected, with no indication of polyhydride phases. The results of these experiments were similar to previously reported attempts^29^, however, we were able to identify Li and Na metals up to 35 GPa, and 50 GPa, respectively, without complete transformation to the monohydride form. To overcome possible kinetic barriers to the formation of polyhydrides, we performed laser-heating experiments on these samples. For Li in hydrogen we were able to perform a few experiments above 50 GPa with laser heating up to 1,900–2,000 K, in which only the monohydride of Li (LiH) was formed. We were not able to detect any polyhydrides of Li under these conditions. Similar measurements for Na in hydrogen at 32 GPa yielded a significant enhancement of the XRD signal from NaH. Further heating of Na and NaH in H_2_-saturated environment to ∼2,100 K produced a laser flash that resulted in sample changes (runaway material forming a ring centred at the flash position—see inset in Fig. 3), indicating the onset of chemical reactions. The Raman spectra collected from temperature quenched sample within the reacted area showed the formation of a new material with two additional vibron peaks ∼4,000 cm^−1^, one of them softer than the pure H_2_ vibron, and the other one harder (Fig. 2; low-frequency Raman spectra are shown in the Supplementary Fig. 1). However, we were unable to detect a reliable XRD signal from the very tiny sample reaction area. We repeated the laser-heating experiment with a NaH sample loaded in the DAC with H_2_ and Au fragments for measuring pressure and for better coupling to the laser during heating. This experiment produced large amounts of a new phase after laser heating at 30 GPa (an example of the XRD pattern obtained after pressure increase to 40 GPa is shown in Supplementary Fig. 2). As the temperature was increased above 2,100 K the thermal runaway resulted in a very bright flash (avalanche) saturating the detector. From the brightness of the heating spot, we estimated the temperature to be in the range of 4,000–6,000 K. We did not attempt to repeat heating due to the risk of breaking the diamonds but saved the sample for further characterization. After heating we could clearly see the change in the sample shape, indicating the sample transport within the laser-heated reaction area of ∼20 μm in diameter.

The newly synthesized phases were characterized by XRD and Raman measurements in the pressure range from 18 to 50 GPa. Decompression of the DAC below 18 GPa resulted in decomposition of the newly formed phase, which was confirmed by the disappearance of their characteristic Raman signatures. These experiments are very challenging since the presence of hydrogen under high-pressure–temperature conditions often leads to diamond breakage. Most of the experiments resulted in failure of the diamond during laser heating; however, we succeeded in producing Na polyhydrides in two runs out of 10, and characterized them using Raman spectroscopy and XRD. The experimental results are described below. Before describing these results, we summarize below our theoretical findings, which differ in a number of aspects from the previous theoretical study of Baettig *et al*.^8^. These differences are crucial for understanding our experimental results.

### Theoretical calculations of sodium polyhydride structures

We searched for low-enthalpy structures using a variety of compositions of Na–H at 50 GPa with the *ab-initio* Random structure searching (AIRSS) method^6^, which has previously been applied to hydrides under pressure^6^,^30^. The calculations used density functional theory^31^,^32^ and the generalized gradient approximation of Perdew, Burke and Ernzerhof for the exchange-correlation functional^33^,^34^. AIRSS was conducted at 50 GPa with the Cambridge serial total energy package (CASTEP) plane-wave code^35^ and ultrasoft pseudopotentials^36^. Further details are provided in the Methods section.

**Figure 1:**
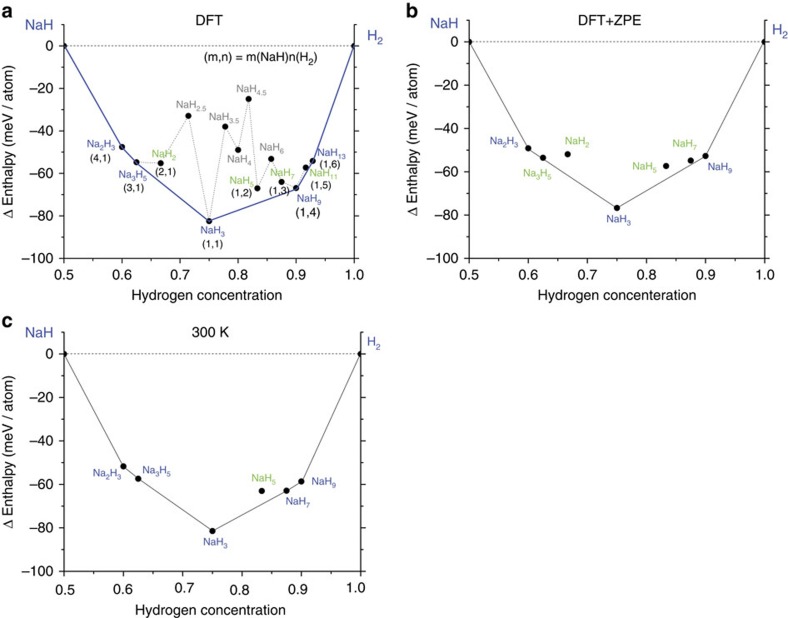
Calculations of stable sodium polyhydride compounds. Convex hull curve of Na–H compounds at 50 GPa with respect to the decomposition (horizontal dashed line) into NaH and H_2_ using (**a**) density functional theory (DFT) (**b**) including ZPE and (**c**) including temperature (300 K). The chemical formula in blue (green) shows predicted stable (metastable) compounds. The (m,n) correspond to compositions in units of NaH and H_2_, respectively. Chemical formulas in black are found to be stable against the decomposition into NaH and H_2_, however, they possess relatively high total energy compared to other stable (meta-stable) phases.

We performed calculations for the structures reported by Baettig *et al*.^8^ and successfully reproduced their data for NaH_7_, NaH_9_ and NaH_11_. We used AIRSS to study other compositions and we found the NaH_3_ phase. This prompted us to extend our searches to lower hydrogen compositions such as NaH_2_, Na_3_H_5_ and Na_2_H_3_. For most compositions, we studied simulation cells containing 1, 2 and 4 formula units, and for NaH_2_ and NaH_3_, we conducted AIRSS on up to 6 formula units. The most stable materials found consisted of H_2_ and NaH structural units. This finding led us to generalize the form of the stable composition to (NaH)_*m*_(H_2_)_*n*_ (Fig. 1). We studied (*m*,*n*) pairs ranging from (4,1) to (1,6). We also tested other compositions such as Na_2_H_5_, Na_2_H_7_ and Na_2_H_9_, but we found them to be unstable with respect to decomposition into nearby stable compositions, as shown in Fig. 1(a). Previous theoretical work suggested that NaH_*n*_ (*n*>6) can be stabilized above 50 GPa (ref. 8). As shown in the convex hull diagram of Fig. 1 at 50 GPa, generally, many combinations of (NaH) and H_2_ can be stabilized. The Na_2_H_3_, Na_3_H_5_, NaH_3_, NaH_9_ and NaH_13_ phases (shown in blue) lie on the convex hull at 50 GPa. In addition, although they are not thermodynamically stable, NaH_2_, NaH_5_, NaH_7_ and NaH_11_ (shown in green) are dynamically stable as demonstrated by the phonon dispersion data (corresponding structures, phonon and electron DOS are shown in Supplementary Figs 3–29 and in the paper of Baettig *et al*.^8^).

The enthalpy differences between the thermodynamically stable phases (blue line) and the dynamically stable phases (green) are only ∼10 meV per atom. We also calculated the nuclear zero-point energy (ZPE) within the harmonic approximation to estimate the effects of vibrations on the total enthalpy. We found a monotonic increase in the ZPE with the fraction of H atoms in the various hydrides ranging from 150 meV per atom in NaH to ∼240 meV per atom in H_2_ (Supplementary Fig. 30).

Figure 1(a) shows that NaH_7_ is not thermodynamically stable but in our calculations including ZPE effect (Fig. 1(b)), we found that NaH_7_ comes within 1–2 meV per atom of being thermodynamically stable. The stability of NaH_7_ relative to other stable phases increases with temperature, and in Fig. 1(c), we show that NaH_7_ eventually becomes a thermodynamically stable phase at 300 K. The temperature corrections for different polyhydride phases are summarized in Supplementary Fig. 31.

### Analysis of synthesized sodium polyhydride phases

**Figure 2:**
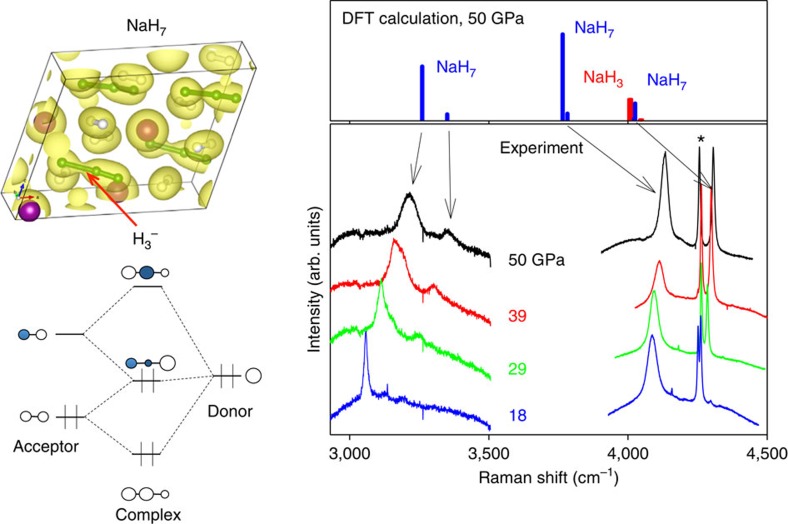
H_3_^−^ complexes in NaH_7_ and Raman spectra of NaH_3_ and NaH_7_. The right panel shows higher-frequency vibrons from H_2_ molecular-type structural units. The left panel shows the structure of NaH_7_, which contains H_3_^−^ complexes. The isosurface is plotted at the level of 0.07 electrons*Å^−3^. One of H_2_ molecules is bonded to a hydrogen atom in the NaH unit with a bond length of *z*=1.25 Å, and they form a H_3_^−^ linear anion in NaH_*x*_ materials with *x*=7. A detailed charge analysis is presented below (Fig. 6). The schematic diagram for H_3_^−^ molecular orbitals (adopted from ref. 48 for I_3_^−^) is also shown. Donor stands for the hydride ion H^−^, and acceptor for the H_2_ unit attached to H^−^. Right panel: Raman spectra of the NaH_7_ sample are shown in the frequency range (3,000–3,500 cm^−1^) typical for vibrons from H_3_^−^ units (indicated in the structure of NaH_7_ as green-yellow dumbells). The Raman response in 4,000–4,300 cm^−1^ region is a mixture of H_2_ vibron modes of NaH_3_ and NaH_7_. The top panel shows the calculated Raman frequencies and intensities for NaH_3_ and NaH_7_. The Raman signal from a pure H_2_ vibron is indicated by an asterisk.

The Raman spectra of the NaH_*n*_ materials synthesized by laser heating (Fig. 2) show a number of features, which are distinct from those of the pure hydrogen within the same sample chamber under the same pressure (50 GPa). New modes that are observed at 4,100 cm^−1^ and 4,200 cm^−1^ bracket the H_2_ vibron at 4,160 cm^−1^ and point to the formation of a new phase containing H_2_ molecules embedded within the sodium polyhydrite crystal structure. Moreover, as shown in Fig. 2, another set of Raman modes appears around 3,200 cm^−1^, suggesting a strongly modified H_2_ species, possibly similar to the predicted H_3_^−^ species or molecules in polyhydrides of Cs (ref. 11) or K (ref. 9). Similar or even lower Raman frequencies are characteristic of dihydrogen moieties observed in transition metal complexes^37^,^38^ and other chemical environments^39^. The low-frequency region of the Raman spectra (Supplementary Fig. 1) also suggests a structure very different from pure hydrogen (for example, ref. 40) and the initial body-centred cubic (bcc) NaH monohydride, which is not expected to have any allowed first order Raman active modes. Indeed, our Raman measurements for unreacted sample regions in the DAC did not produce any Raman signatures of NaH, but indicated the presence of pure solid H_2_, judging from its characteristic vibron and roton bands. The low-frequency Raman spectrum of the newly synthesized material consists of strongly pressure-dependent bands at 200–800 cm^−1^, which we identify as lattice modes in contrast to weakly pressure-dependent rotational modes of pure H_2_. (Supplementary Fig. 1)

**Figure 3:**
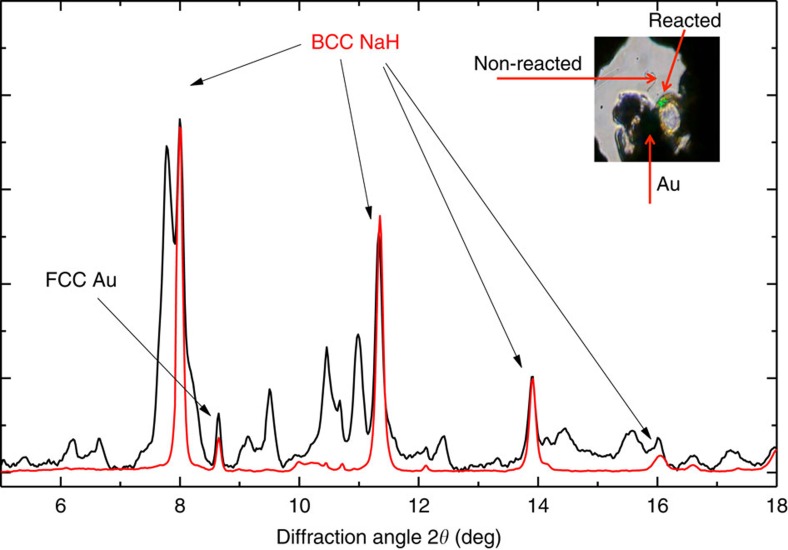
Structural information from XRD measurements. XRD raw pattern (black) of the reacted area of the sample at 50 GPa, containing Bragg peaks from BCC NaH and FCC Au, which are indicated by arrows. The red XRD pattern is from a non-reacted area of the sample containing only BCC NaH and FCC Au. The perfect match of the position of the NaH peaks between the reacted and the non-reacted area justifies our argument about the origin of these peaks. The inset shows a reacted sample, dark sample in a gasket hole is Au+Na. The transparent part is NaH+H_2_, the smaller dark circle with a green laser spot is a reacted area. The darker colour of the reacted area is compatible with a reduced bandgap (∼2 eV) obtained in the DFT calculations (Fig. 6).

**Figure 4:**
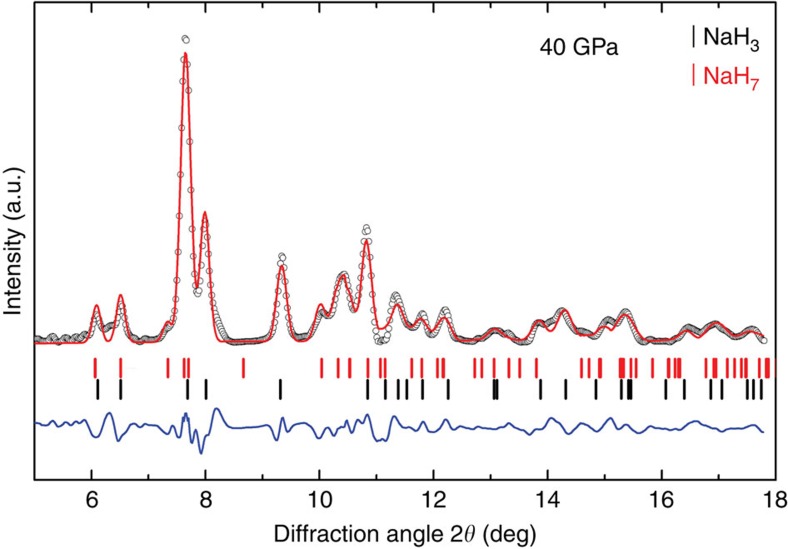
Le Bail refinement for NaH_n_ at 40 GPa. NaH_3_ and NaH_7_ peaks are marked with black and red vertical lines, respectively. The difference between the data and the fit is shown below (blue line).

**Figure 5:**
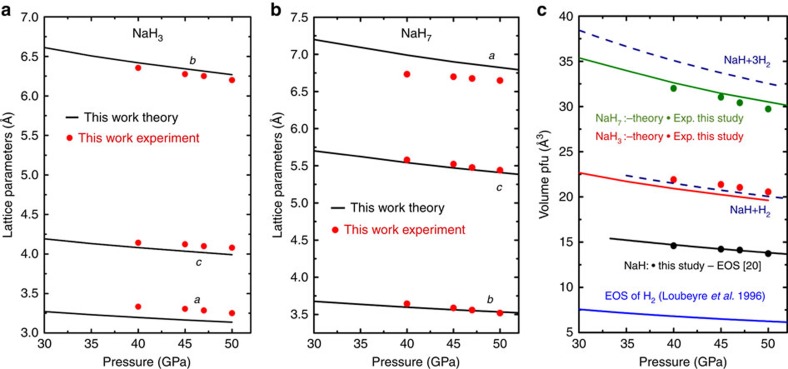
Lattice parameters and equations of state of NaH_3_ and NaH_7_. (**a**) Lattice parameters of NaH_3_ as function of pressure. (**b**) Lattice parameters of NaH_7_ as function of pressure. (**c**) Equations of state (EOS) of NaH_3_, NaH_7_ in comparison with EOS of NaH and H_2_. Experimental data: green, red and black circles, theoretical predictions: green, red and black continuous lines (specified in the figure). EOS of H_2_ is also shown (blue line).

**Figure 6:**
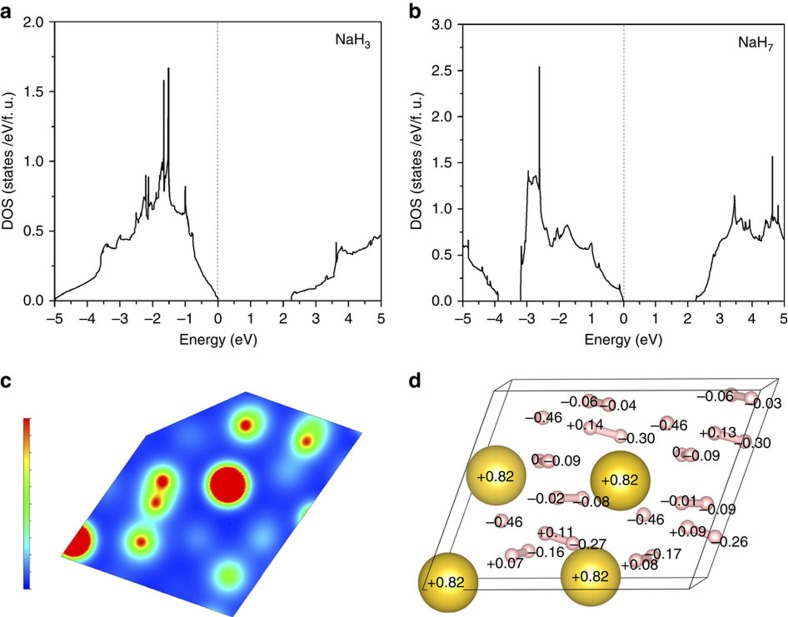
Calculated electronic properties of NaH_3_ and NaH_7_ at 50 GPa. Density of electronic states of NaH_3_ (**a**) and NaH_7_ (**b**). (**c**) A contour plot of H3^−^ unit in NaH_7_. This image shows a charge density contour with a saturation level of 0.3 electrons Å^−3^ (which is much higher than 0.07 of the isosurface plot in Fig. 2). An equi-charge density level of H3^−^ unit is evident from the plot, which was prepared for the Miller indices (1 2 −1). (**d**) Bader analysis showing excessive charge of individual atoms in NaH_7_. Na cations have a charge +0.82 and ionic linked hydride H has a charge of −0.46. H_2_ molecules with higher vibron frequencies have less polarized charges (they form pairs with charges −0.06 & −0.04, −0.06 & −0.03, 0 & −0.09, −0.02 & −0.08, +0.07 & −0.16). However, the H_2_ molecules which are linked to the hydride ion H(−0.46) are highly polarized (+0.14 & −0.30, +0.13 & −0.30, +0.11 & −0.27, +0.09 & −0.26).

Figures 3 and 4 show an XRD pattern of a new material at 40 GPa. XRD data were also obtained away from the reacted area at each pressure (see inset to Fig. 3). Three different ‘families' of reflections from different phases were observed to coexist in the XRD patterns of the reacted area: (i) the unreacted bcc NaH (ambient pressure face-centred cubic (fcc) NaH transforms to bcc at 29 GPa (ref. 41)), (ii) the fcc Au used as a pressure marker and as a laser absorber and (iii) the synthesized NaH_*n*_. To fully identify the reflections from the synthesized NaH_*n*_, we performed a detailed comparison of the XRD patterns on and away from the reacted area. A typical example is shown in Fig. 3. The positions of all reflections attributed to NaH and Au are in full agreement with the known diffraction peaks of bcc NaH (ref. 41) and fcc Au, implying the absence of a chemical reaction between Au and H. The reflections of bcc NaH and Au have then been subtracted when performing the final structural refinement of the NaH_*n*_ phases (Supplementary Fig. 2). This has been performed via a Rietveld refinement only for bcc NaH and fcc Au with a subsequent subtraction of the refined peaks from the raw patterns. After all reflections not belonging to the synthesized NaH_n_ have been successfully identified we compared the calculated XRD patterns of the predicted stable structures with the observed ones. Full indexing-refinement of the observed reflections, without the use of the predicted phases as candidates, is very difficult for a variety of reasons. First, the XRD intensity depends almost exclusively on the positions of the Na atoms. Second, the large number of observed peaks suggests a low-symmetry unit cell. Finally, the texture of the two-dimensional images of the XRD data suggests a mixture of phases. Based on this analysis, we find that NaH_3_ is the predominant phase of the synthesized material (Fig. 4). Indeed, all the main reflections can be indexed with the orthorhombic *Cmcm* NaH_3_ cell. Moreover, the experimentally determined lattice parameters and cell volume (at 40 GPa: *a*=3.332 Å, *b*=6.354 Å and *c*=4.142 Å with *V*_pfu_=21.93 Å^3^) of NaH_3_ are in full agreement with the theoretical predictions (Fig. 4). However, there are a few reflections that cannot be indexed with the NaH_3_ cell. For hydrogen contents lower than in NaH_5_, the phonon density of states has two well-separated bands, below 1,500 cm^−1^ for Na–H interactions and around 4,000 cm^−1^ for H_2_ vibrations. At higher hydrogen concentrations, we found the formation of other intermediate frequency bands near 3,200 cm^−1^. Having in mind that NaH_*n*_ phases (with *n*<7) cannot support the existence of Raman modes at 3,200 cm^−1^ (Supplementary Figs 5, 6, 8, 9, 11, 12, 14, 15, 17, 18 and 21) we have to include phases with *n*>6 (refs 7, 9) in our analysis. From the various phases only the monoclinic *Cc* NaH_7_ phase shows reasonable agreement with the observed patterns. Indeed, some of the main observed reflections can only be indexed with the NaH_7_ phase with experimental lattice parameters *a*=6.99 Å, *b*=3.597 Å, *c*=5.541 Å and *β*=69.465° (theoretical values *a*=6.732 Å, *b*=3.643 Å, *c*=5.577 Å and *β*=69.36°) at 40 GPa. With the use of both phases, NaH_3_ and NaH_7_, we have successfully indexed all observed reflections of the synthesized mixed-NaH_*n*_ material (Fig. 4). The experimental and theoretical lattice parameters and volume are summarized as a function of pressure in Fig. 5. Notably, while the experimental volumes of NaH_3_ and NaH+H_2_ are very close, the volume of NaH_7_ is significantly lower than that of NaH+3H_2_. The PV term of NaH_3_ is practically the same (given the experimental error in both the reported EOS of NaH and H_2_) with that of NaH+H_2_. There is very good agreement between observed and theoretically predicted relative intensities of Bragg reflections. However, a refinement of the positional parameters was not possible due to the ‘spotty' XRD rings. Finally, Fig. 6 provides some details of the electronic structure of new phases as follows from the theoretical analysis. The electronic density of states is compatible with insulating phase for both materials, with a reduced bandgap slightly larger than the value of 2 eV obtained from a DFT calculation. It is well known that standard DFT method underestimates the bandgaps of most semiconductors and thus it is expected that the real band gap in NaH_3_ and NaH_7_ could be larger than the calculated one. We calculated metallization pressures for NaH_3_ of about 250 GPa, which are similar to those predicted for higher polyhidrides in ref. 8. For NaH_7_ we found that electronic density contours clearly indicate formation of H3-units—Fig. 6(c,d).

## Discussion

The Raman and XRD data point to the formation of Na polyhydrides in the predicted stability range (above 20 GPa). While we were unable to isolate a single well-defined polyhydride phase, the data analysis strongly supports the existence of several phases (NaH_3_ and NaH_7_, and possibly higher polyhydrides) in the reacted sample. Most of the theoretically predicted stable Na polyhydride phases have low-symmetry structures, which are extremely difficult to characterize by XRD from the small samples available in the laser-heated region. While prolonged laser heating at well-defined *P-T* conditions may be beneficial for growing a single-phase sample, such experiments are still inaccessible due to the high reactivity of hot hydrogen with diamond anvils.

Notably, Raman spectroscopy provided a more sensitive tool than XRD for characterizing the formation of small amounts of low-Z polyhydride materials. Based on the results of theoretical calculations, we found that the Raman bands observed experimentally near 3,200 cm^−1^ can be assigned to an extended hydrogen molecular H_2_ unit with an intramolecular length *d* of ∼0.82 Å. This H_2_ molecule is linked to a hydrogen atom in the NaH unit with a distance of *z*=1.25 Å by sharing valence electrons (Fig. 6(c,d)), and they form a H_3_^−^ linear anion in NaH_*x*_ materials with *x*=7 (Figs 2 and 6). It was suggested that pressure can induce a linear geometry for H_3_^−^, which has four electrons, but a triangular geometry for H_3_^+^, which has two electrons^20^; recent confirmation of these simple chemical arguments was provided by a careful theoretical study of heavy alkali-metal hydrides under pressure predicted to form linear H_3_^−^ in KH_5_. To gain further insights into H_3_^−^ anion formation in NaH_7_, we analysed the charge density of NaH_3_ and NaH_7_ using Bader analysis (Fig. 6). The calculations confirmed the highly ionic nature of the NaH unit in each polyhydride: the net charges on Na and H in the NaH unit are+0.79/+0.82 and −0.65/−0.47 in NaH_3_/NaH_7_, respectively, indicating that a significant portion of the electron density is donated to the H_2_ molecules in NaH_7_. In fact, the H3-anion in NaH_7_ has an excess of −0.63 electrons and accordingly, H_2_ in H3-anion possesses −0.16 e, which leads to the elongation of the H_2_ bond.

*Ab initio* phonon calculations give information on the dynamical stability of the phases. The stability region of NaH_7_ was predicted^8^ to be 25–100 GPa which is consistent with our experiments. All lattice and vibron modes of the polyhydrides increase monotonically in frequency with pressure up to 50 GPa. Our theoretical calculations show dynamical stability and structural stability of predicted phases, including NaH_3_ and NaH_7_.

In summary, we synthesized polyhydrides of Na in a laser-heated DAC at pressures above 30 GPa and temperatures above 2,000 K. We also performed detailed theoretical studies and found new stable phases of Na polyhydrides. One of these phases, NaH_3_, provides a good match to the XRD patterns collected from the reacted region. However, the x-ray patterns also suggest the existence of higher polyhydrydes (NaH_n_, *n*≥7), which is supported by the analysis of the Raman spectra in the 3,200 cm^−1^region. Notably, higher polyhydrides of sodium appear to stabilize the H_3_^−^ unit predicted for other, heavier alkali metals^18^. Polyhydrides of alkali metals provide a new class of materials with pressure-stabilized multicenter (3 center—4 electron) bonds for future investigation. Polyhydrides may provide chemical means to pre-compress hydrogen molecules and facilitate the creation of metallic superconducting hydrogen at reduced pressures. The possibility of metastable phases should be carefully explored in future studies, since the new polyhydrides may be implemented as hydrogen storage materials with hitherto unexplored physical and chemical properties.

## Methods

### High-pressure experiments

We have studied the formation of Li and Na polyhydrides in a DAC at pressures up to 70 GPa with laser heating to 2,000 K and higher temperatures. The experiments were performed in a symmetric DAC (ref. 42). The samples of Li, Na, LiH and NaH were loaded, along with small fragments of Au, in a glove box with controlled atmosphere (<1 p.p.m. of oxygen). According to recent experimental^43^ and theoretical^44^ results, no chemical reaction is expected between Au and H_2_, up to the highest pressure of this study. Each sample was sealed in a DAC inside a glove box, and transferred to a gas-loading apparatus, where a H_2_ pressure of ∼200 MPa was created. The DAC was opened under the H_2_ pressure to let the gas in, resealed and then taken out for further high-pressure experiments.

XRD measurements and on-line laser heating were performed at the Sector 13 (GSECARS), Advanced Photon Source at the Argonne National Laboratory^45^. The DAC was cooled below 200 K with a nitrogen jet from Cryostream-type unit manufactured by Oxford Cryosystems.

Raman measurements were performed using off-line custom-made Raman system at GSECARS, the data were taken with Ar ion laser excitation (wavelength 514.5 nm).

### Theoretical calculations

We used the CASTEP plane-wave-basis set cutoff energy of 1,000 eV and a Brillouin-zone integration grid of spacing 2π × 0.05 Å^−1^. Phonon calculations were performed with density functional perturbation theory using the Quantum Espresso code^46^ with a kinetic energy cutoff of 70 Ry. The BZ integrations in the calculations were performed using Monkhorst–Pack meshes^47^. We refer to meshes of k-points for electronic structure calculations and meshes of q-points for phonons. The phonon calculations used 24 × 24 × 24 k-points mesh and 8 × 8 × 8 q-points mesh for the most studied Na–H compounds and 12 × 12 × 12 k-points with a 6 × 6 × 6 q-points mesh is used for relatively larger unit-cell compounds (Na_2_H_3_ and Na_3_H_5_).Further details regarding the theoretical calculations are available in the Supplementary Methods.

### Data availability

The authors declare that most of the data supporting the findings of this study are available within the article and its Supplementary Information Files. Any additional relevant data are available from the corresponding author on request.

## Additional information

**How to cite this article:** Struzhkin, V. V. *et al*. Synthesis of sodium polyhydrides at high pressures. *Nat. Commun.* 7:12267 doi: 10.1038/ncomms12267 (2016).

## Supplementary Material

Supplementary Information Supplementary Figures 1-31 and Supplementary Methods.
