# Supplementary material for: Synthesis of sodium polyhydrides at high pressures
Source: Nat Commun. 2016 Jul 28;7:12267. doi: 10.1038/ncomms12267 (PMC4974473; doi:10.1038/ncomms12267)
Supplement: Supplementary Information — Supplementary Figures 1-31 and Supplementary Methods. [file ncomms12267-s1.pdf]

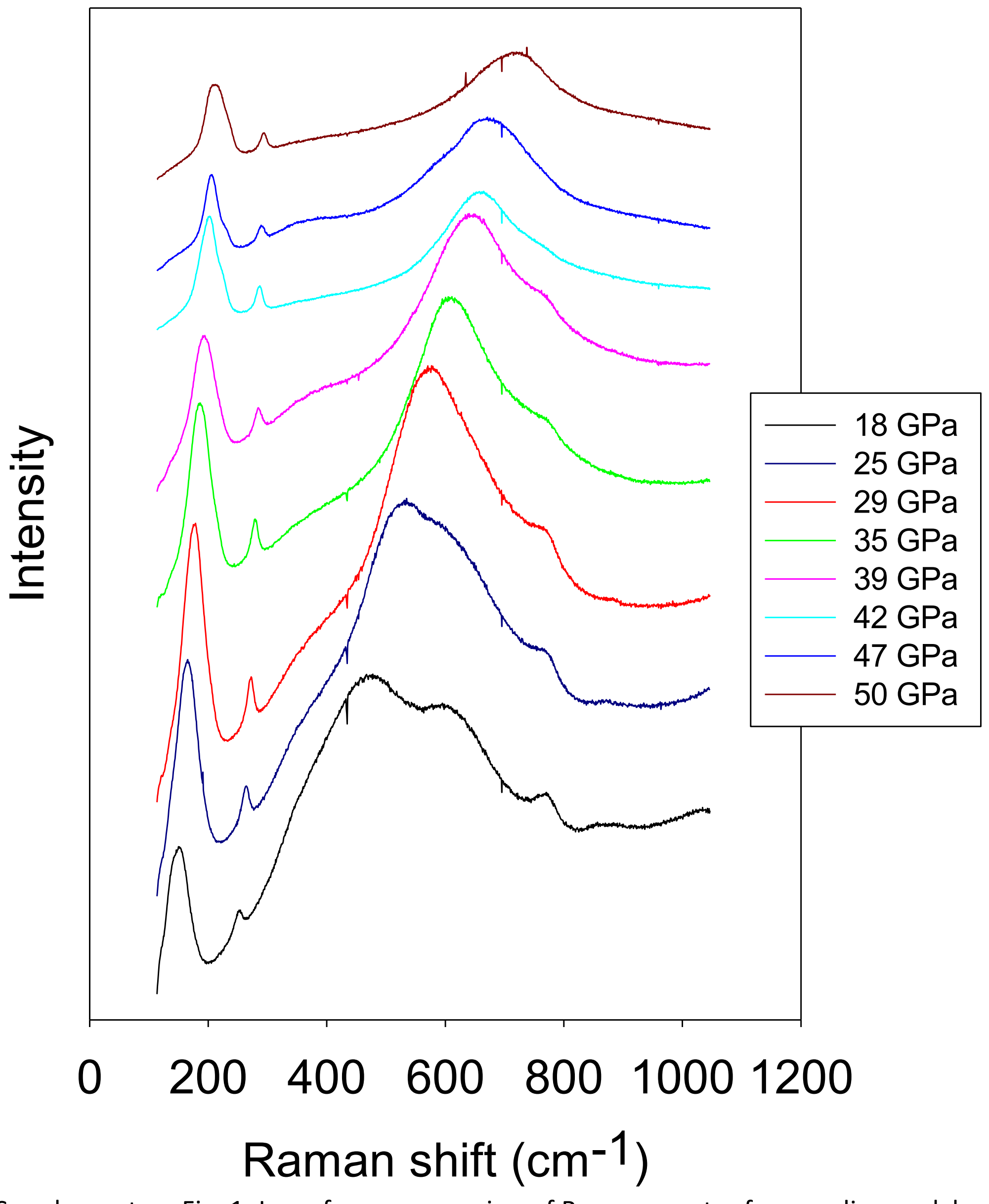

Supplementary Fig. 1. Low -frequency region of Raman spectra from sodium polyhydride sample.

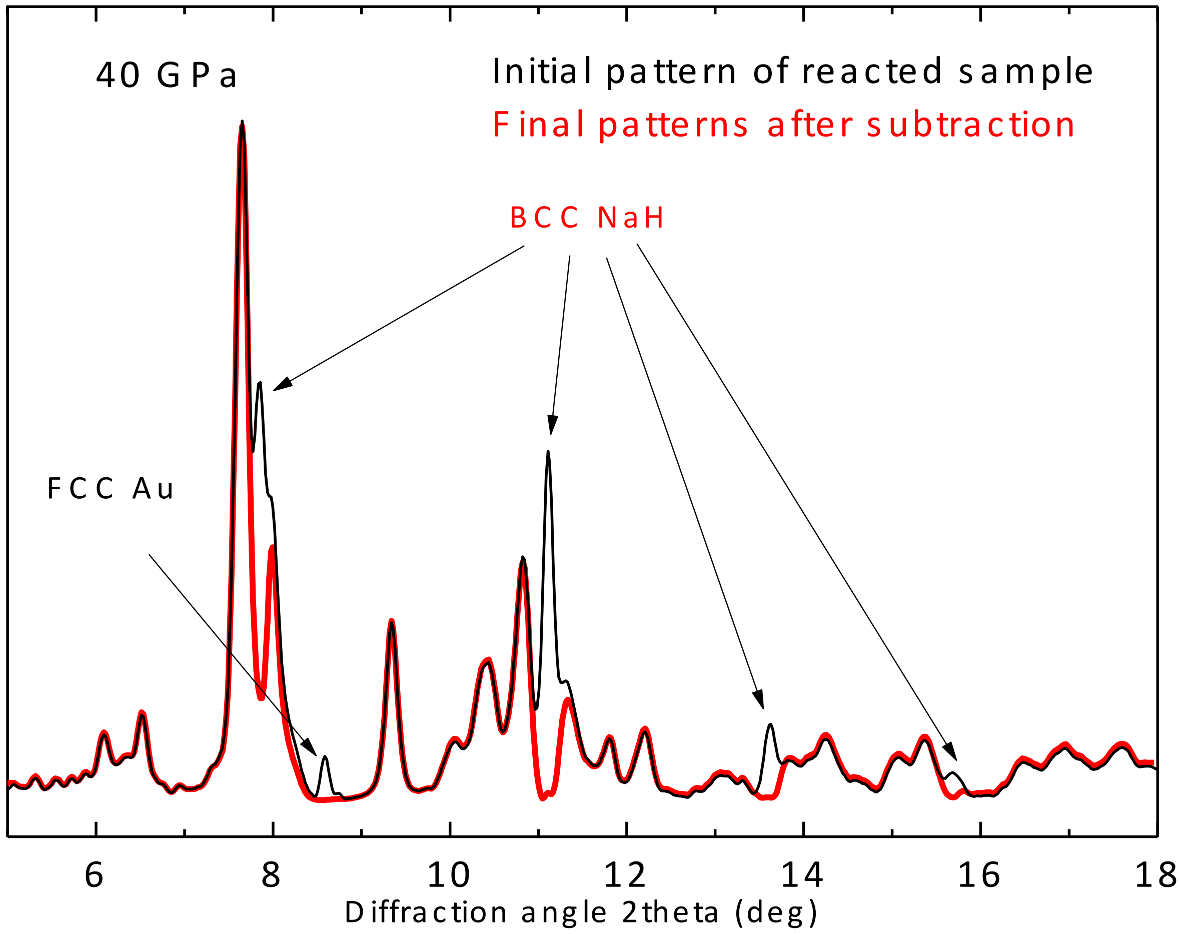

Supplementary Fig 2. X-ray diffraction pattern pattern (black) of the reacted area of the sample at 40 GPa.

Bragg peaks from BCC NaH and FCC Au are indicated by arrows. Final pattern is shown in red)color, after the subtraction of the refined peaks of NaH and Au.

[

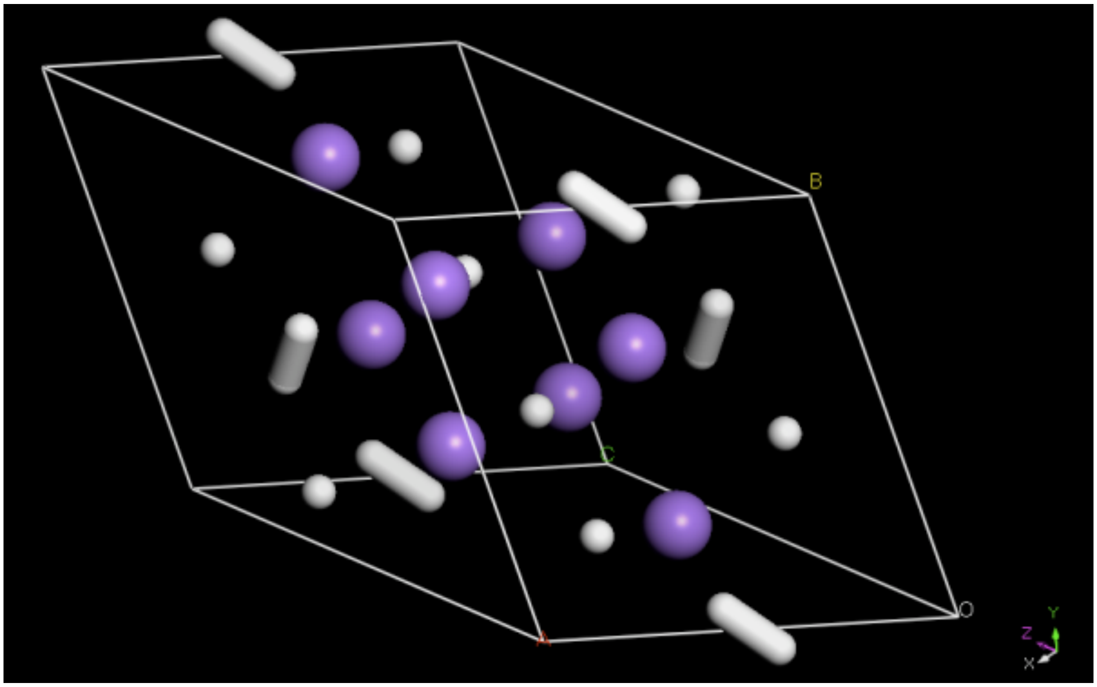

Supplementary Fig. 3. Structural unit of Na_2H_3.

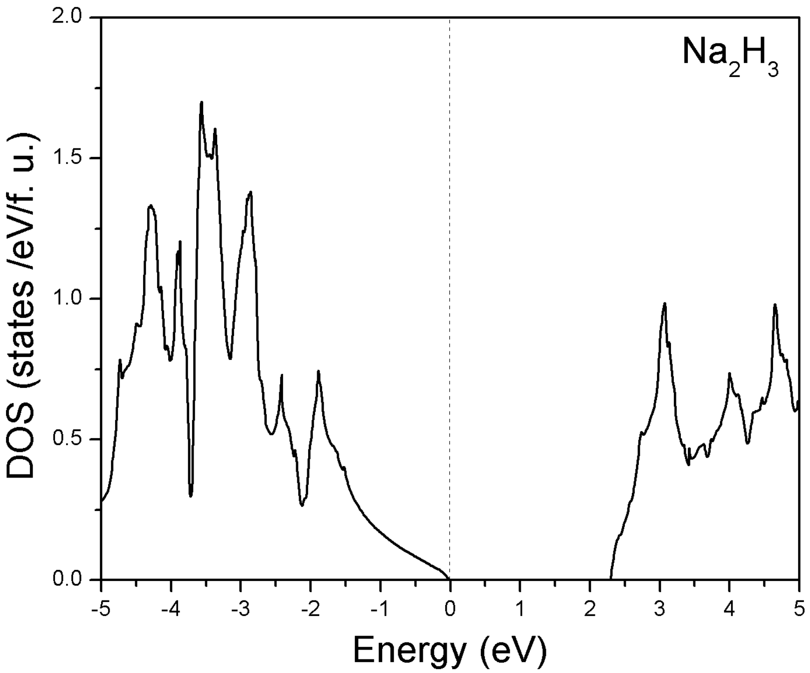

Supplementary Fig. 4. Electron density of states (DOS) for Na_2H_3.

The vertical dashed line shows the Fermi level.

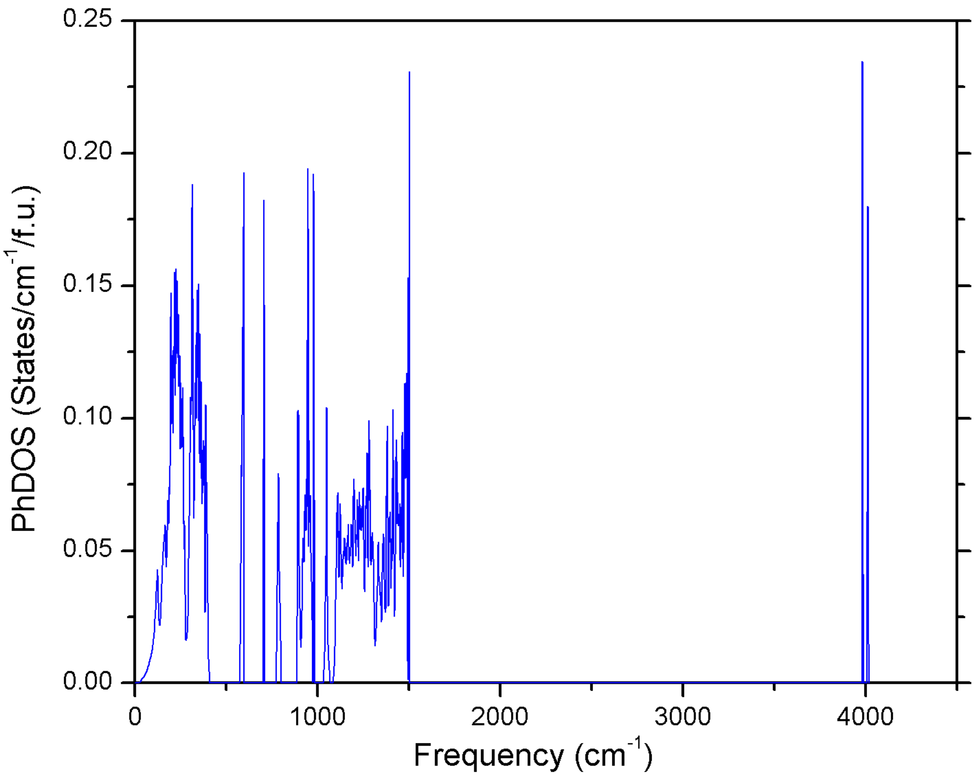

Supplementary Fig. 5. Phonon density of states (DOS) for Na_2H_3

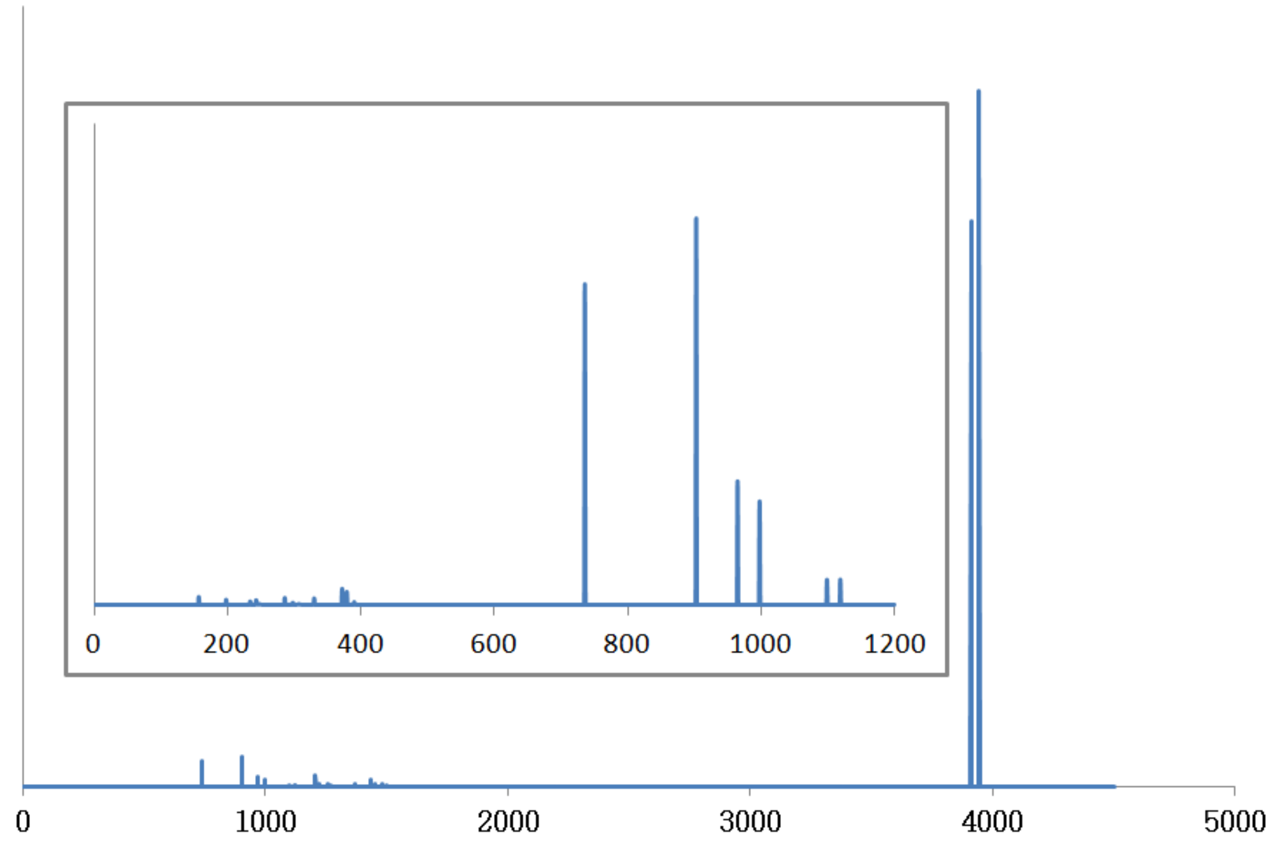

Supplementary Fig. 6. Raman Intensity calculated for Na_2H_3.

Horizontal axis in units of cm^{-1}

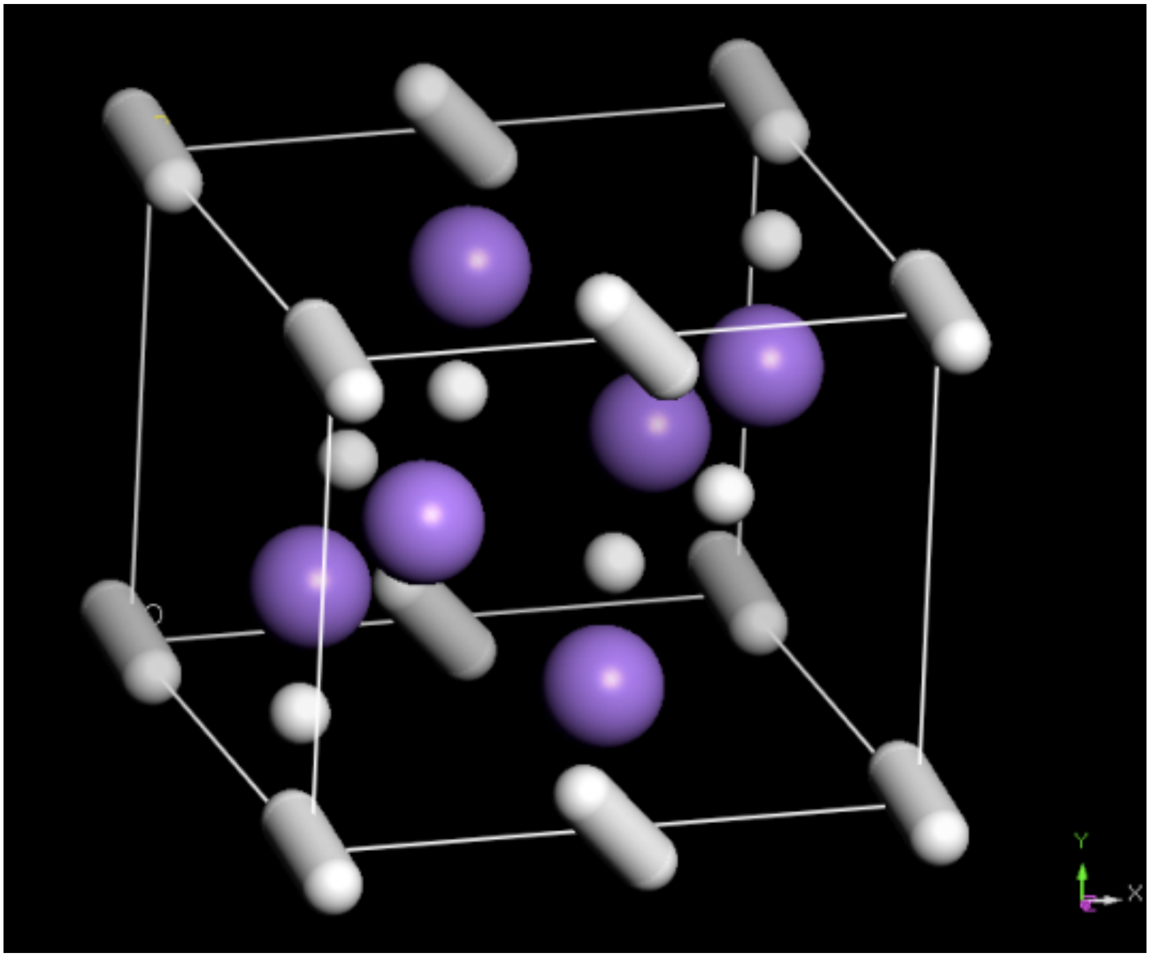

Supplementary Fig. 7. Structural unit of Na_3H_5.

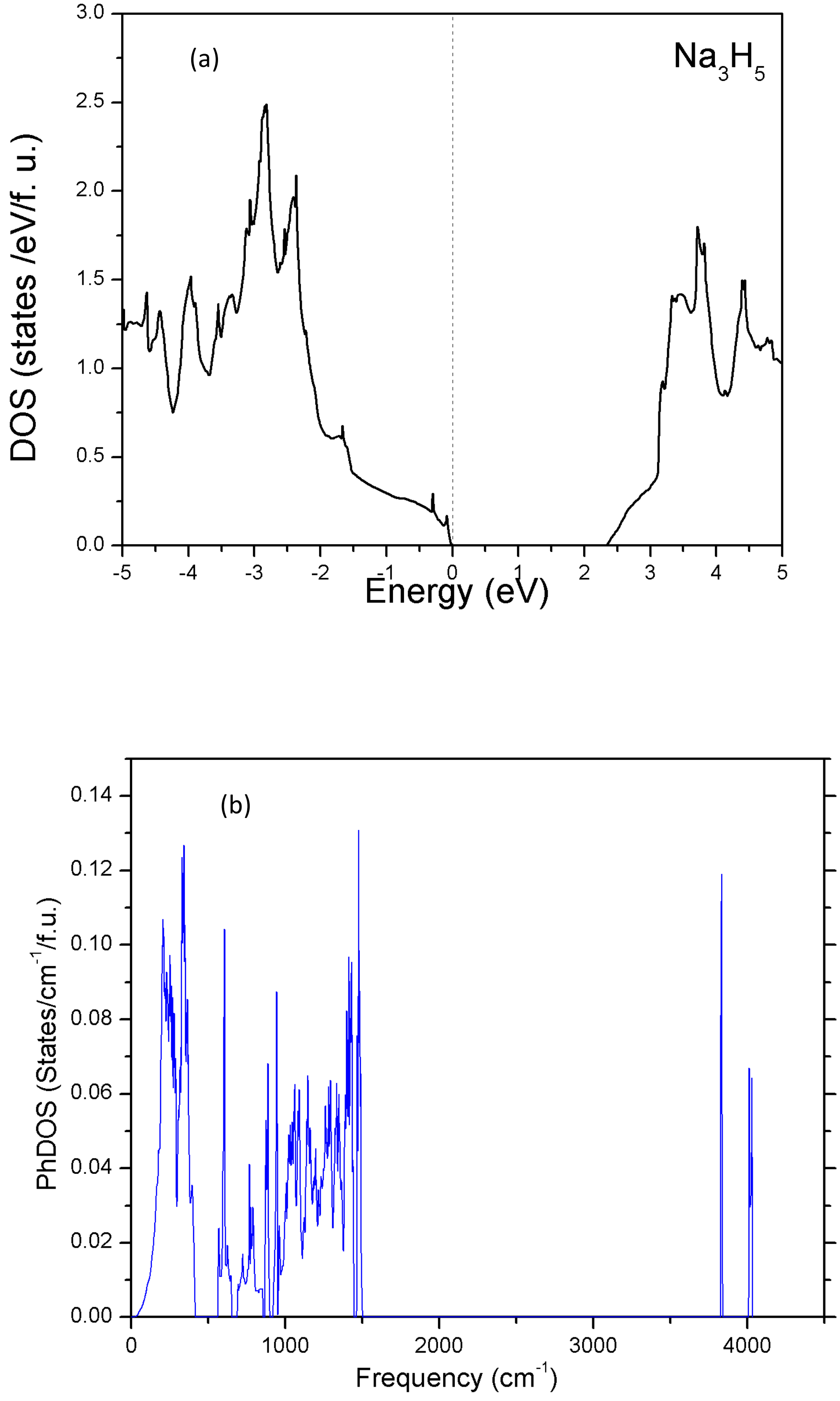

Supplementary Fig. 8. Electron (a) and phonon (b) DOS for Na_3H_5.

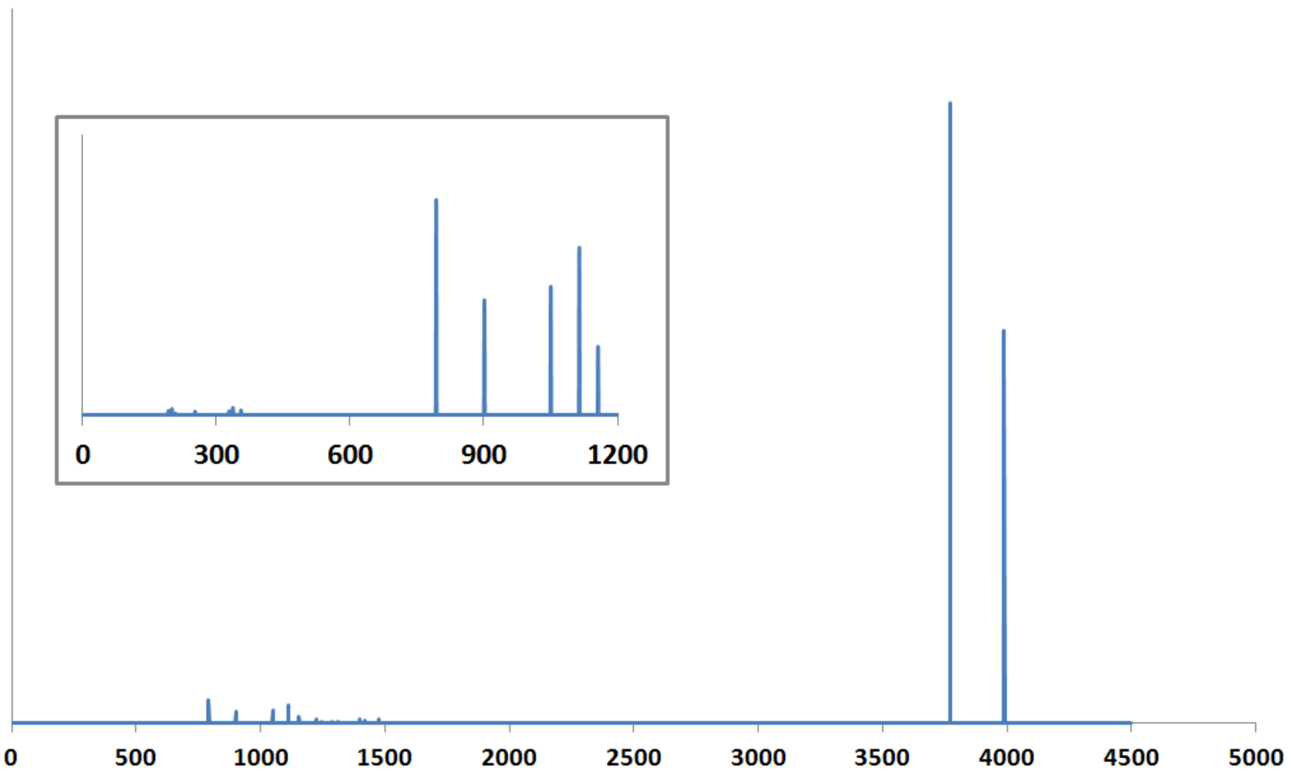

Supplementary Fig. 9. Raman Intensity calculations Na_3H_5, horizontal axis in units cm^{-1}

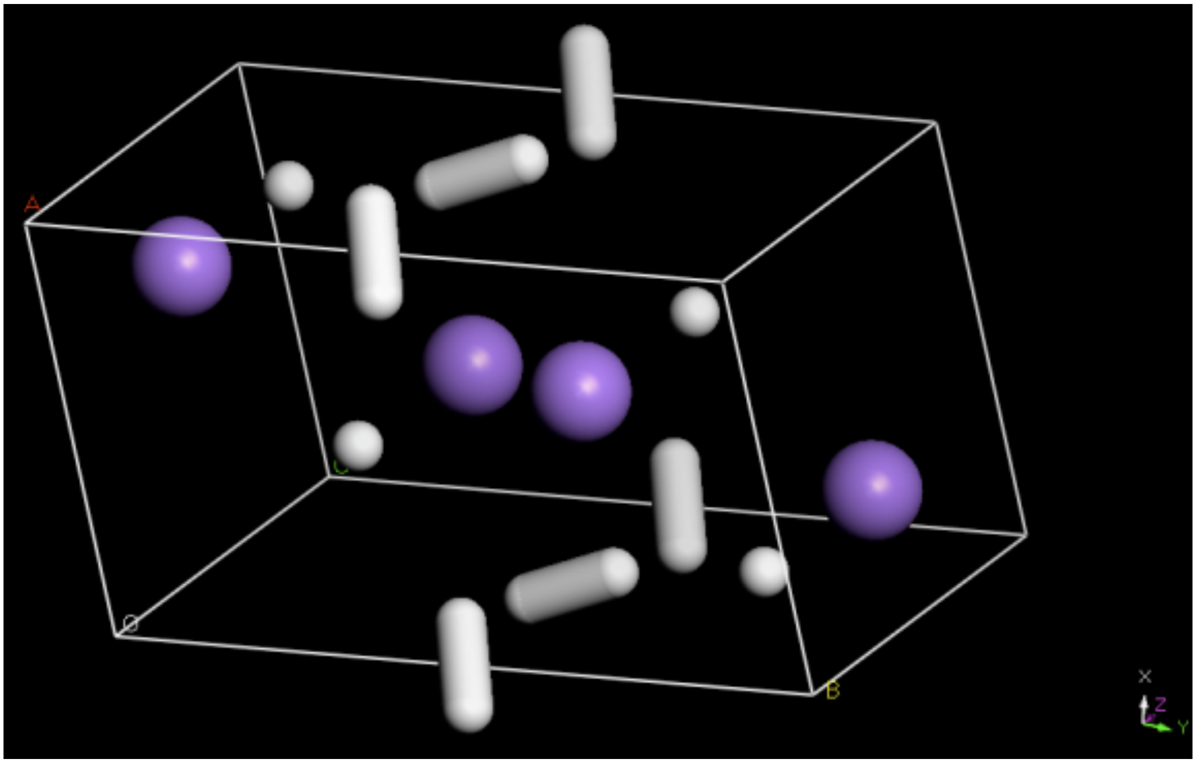

Supplementary Fig. 10. Structural unit of NaH_2.

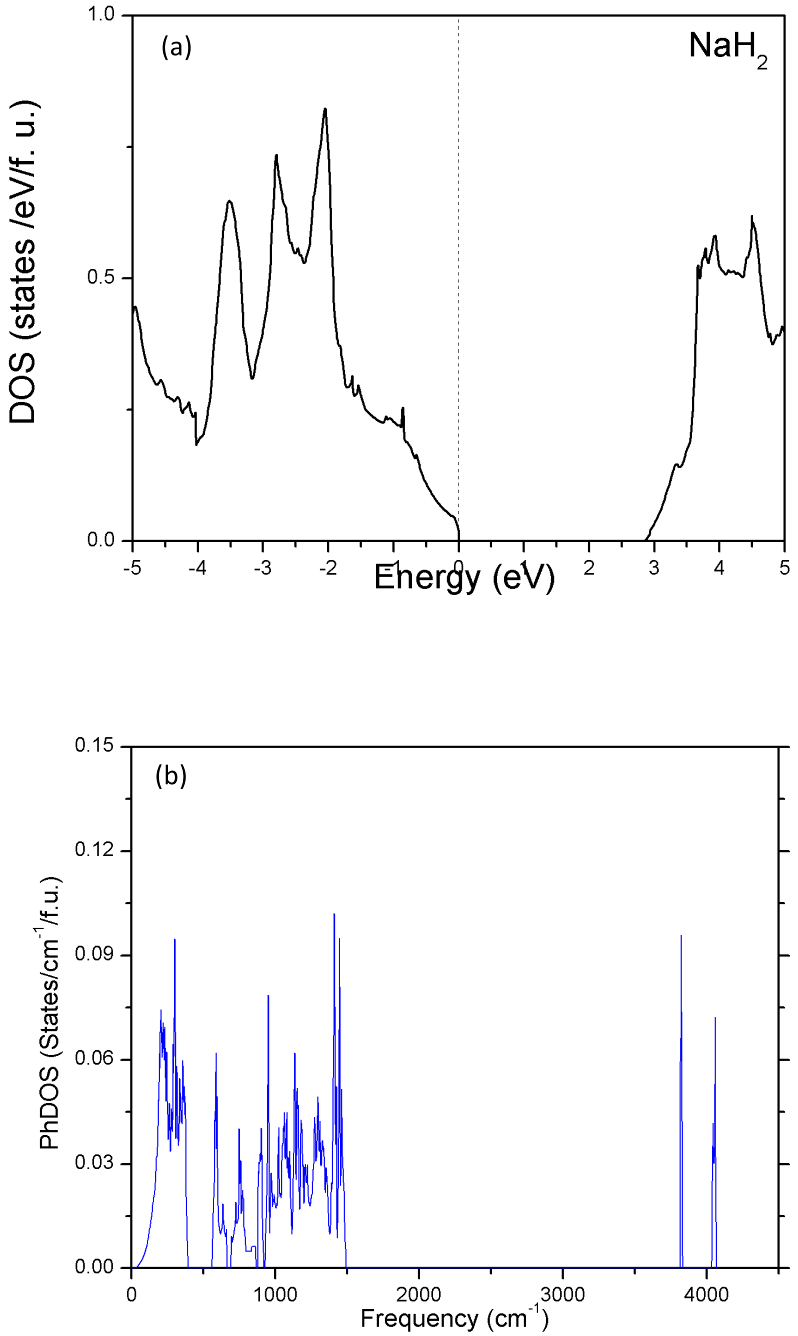

Supplementary Fig. 11. Electron (a) and phonon (b) DOS of NaH_2.

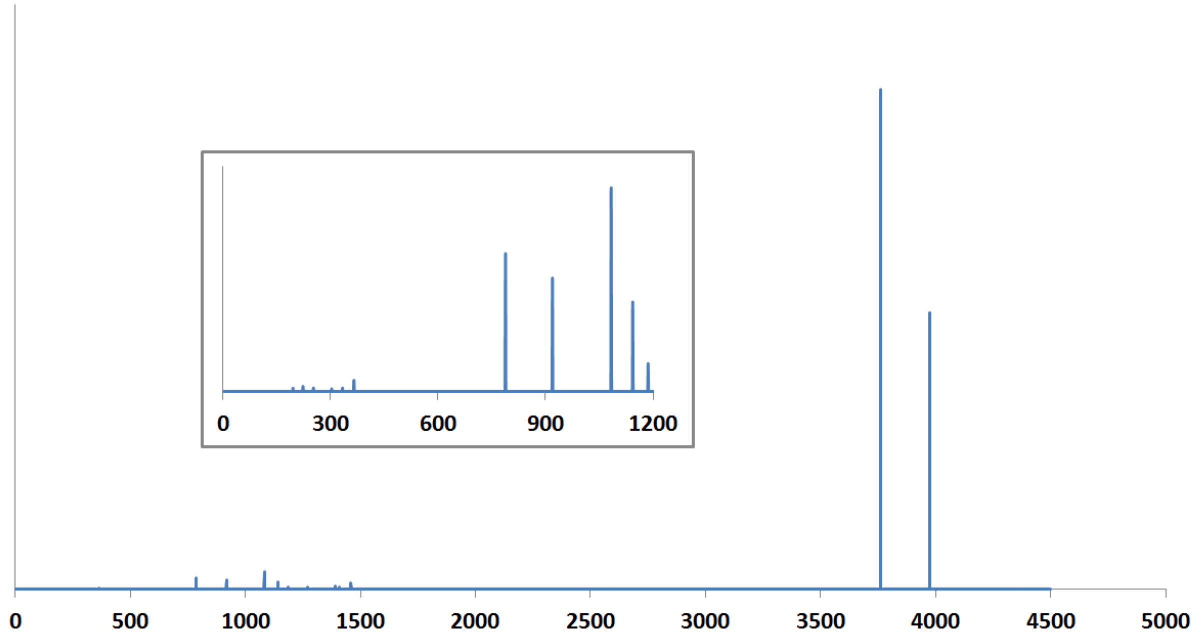

Supplementary Fig. 12. Raman intensity, horizontal axis in cm^{-1} for NaH_2

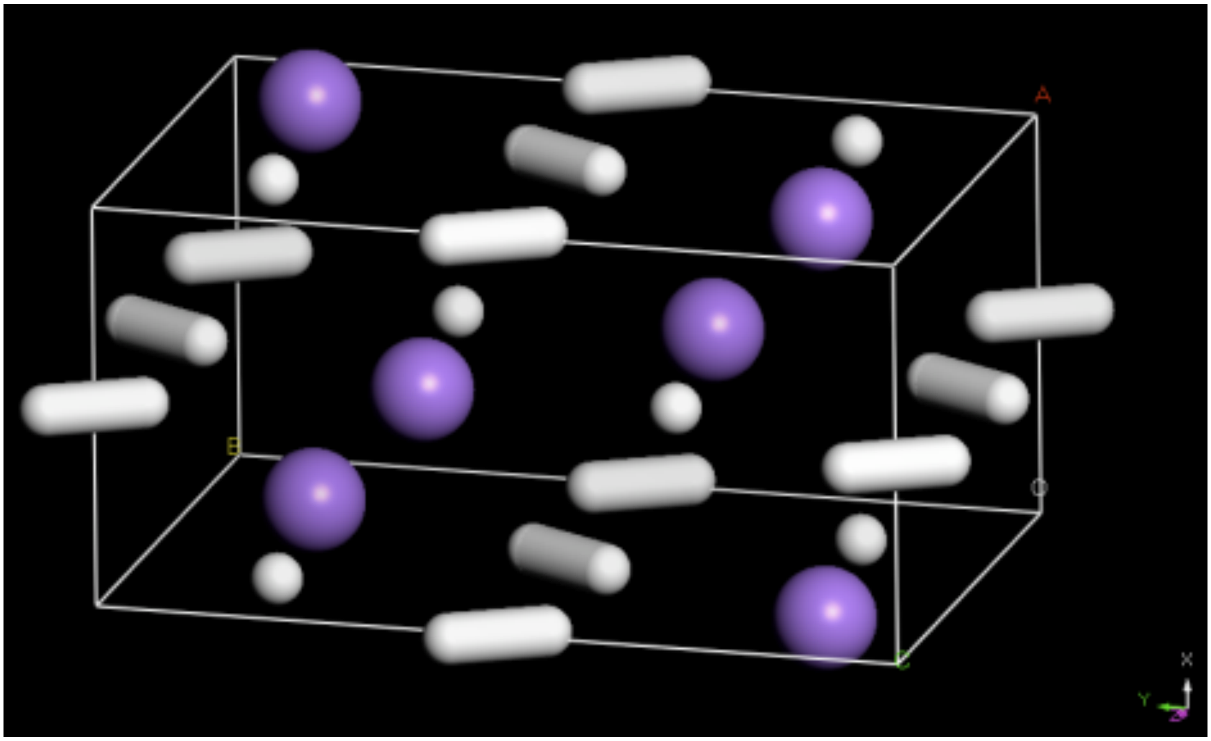

Supplementary Fig. 13. Structural unit of NaH_3.

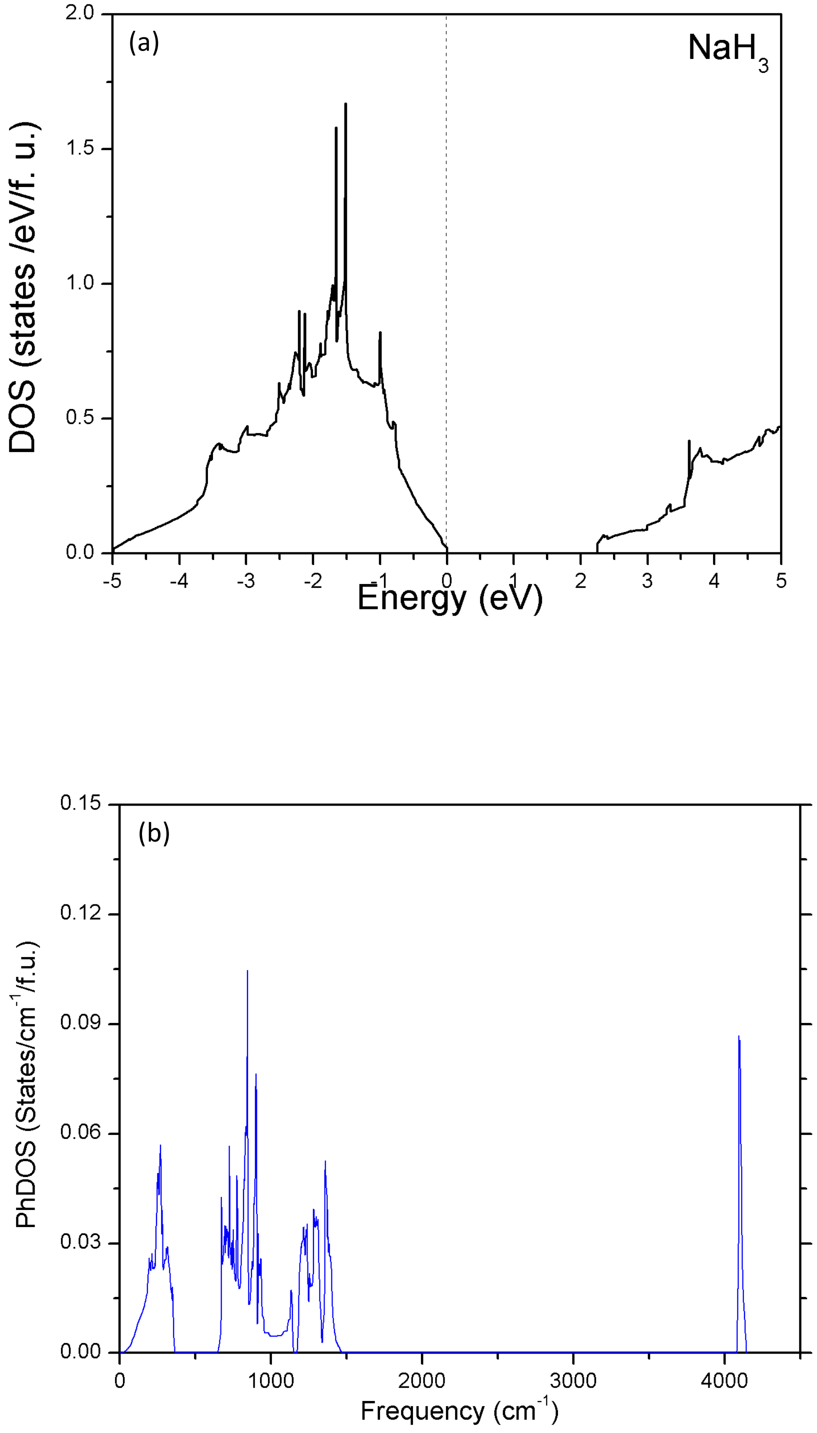

Supplementary Fig. 14. Electron (a) and phonon (b) DOS for NaH_3.

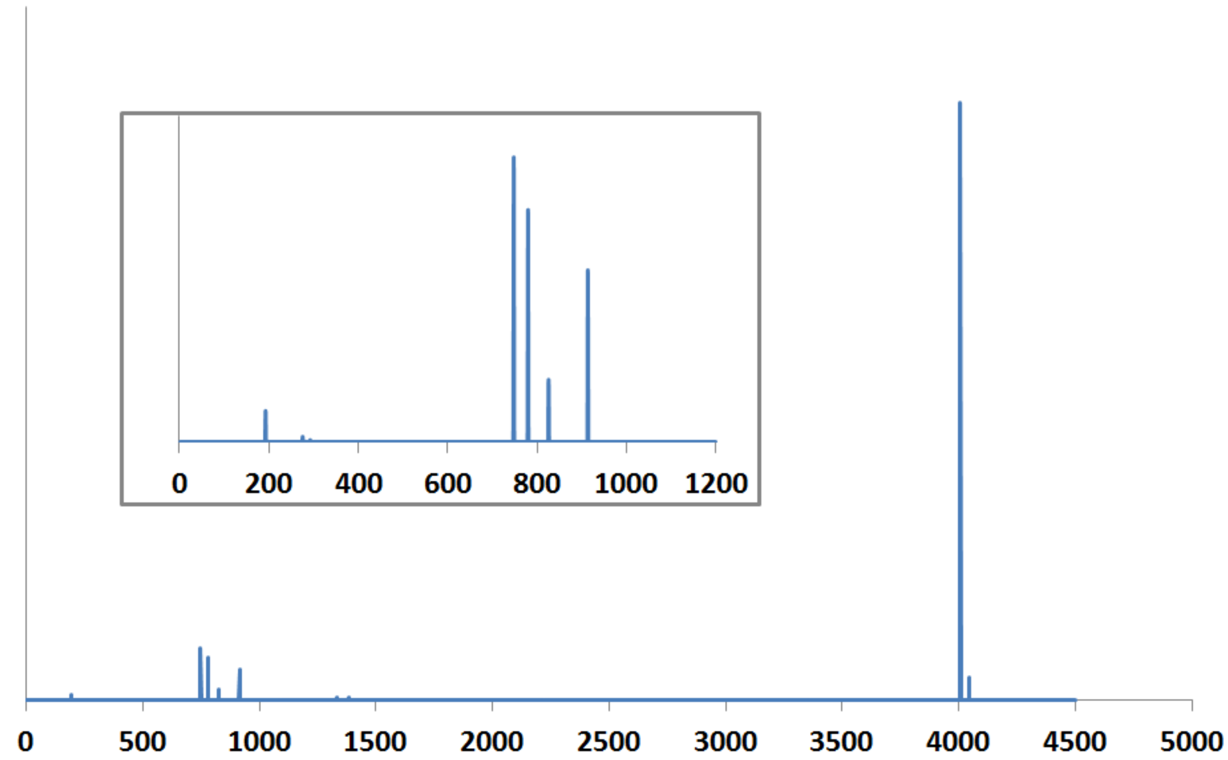

Supplementary Fig. 15. Raman intensity calculated for NaH_3 .

Horizontal axis in units of cm^{-1}.

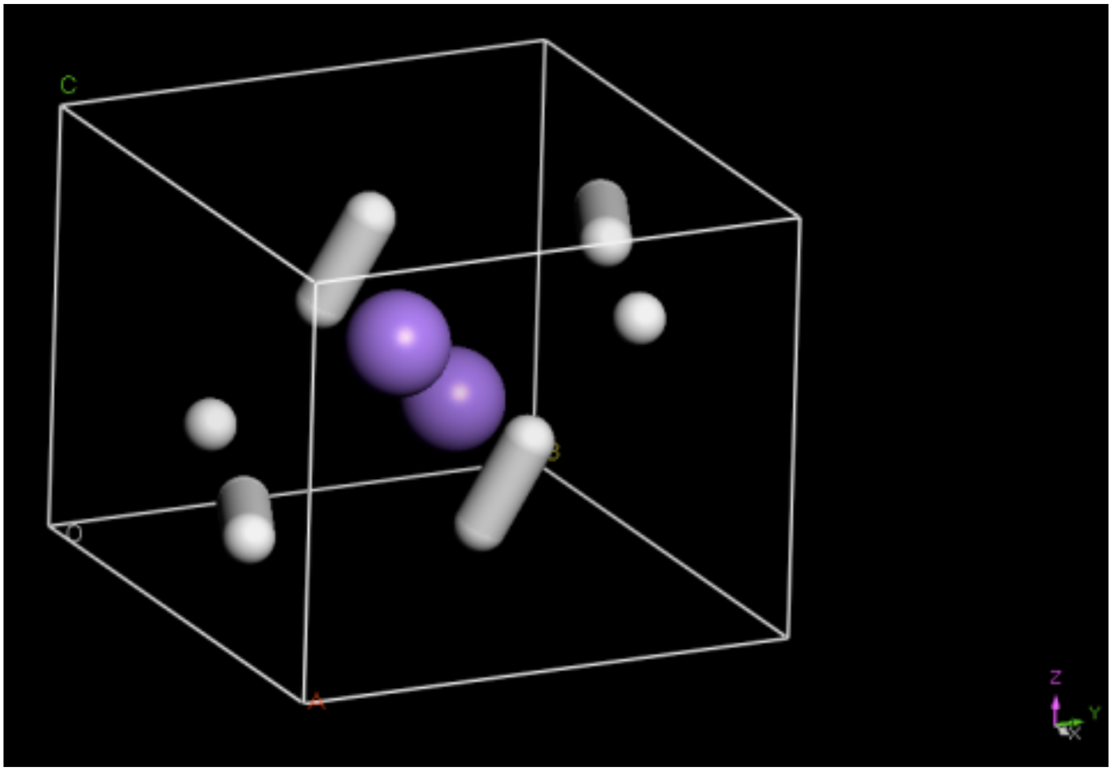

Supplementary Fig.16. Structural unit of NaH_5.

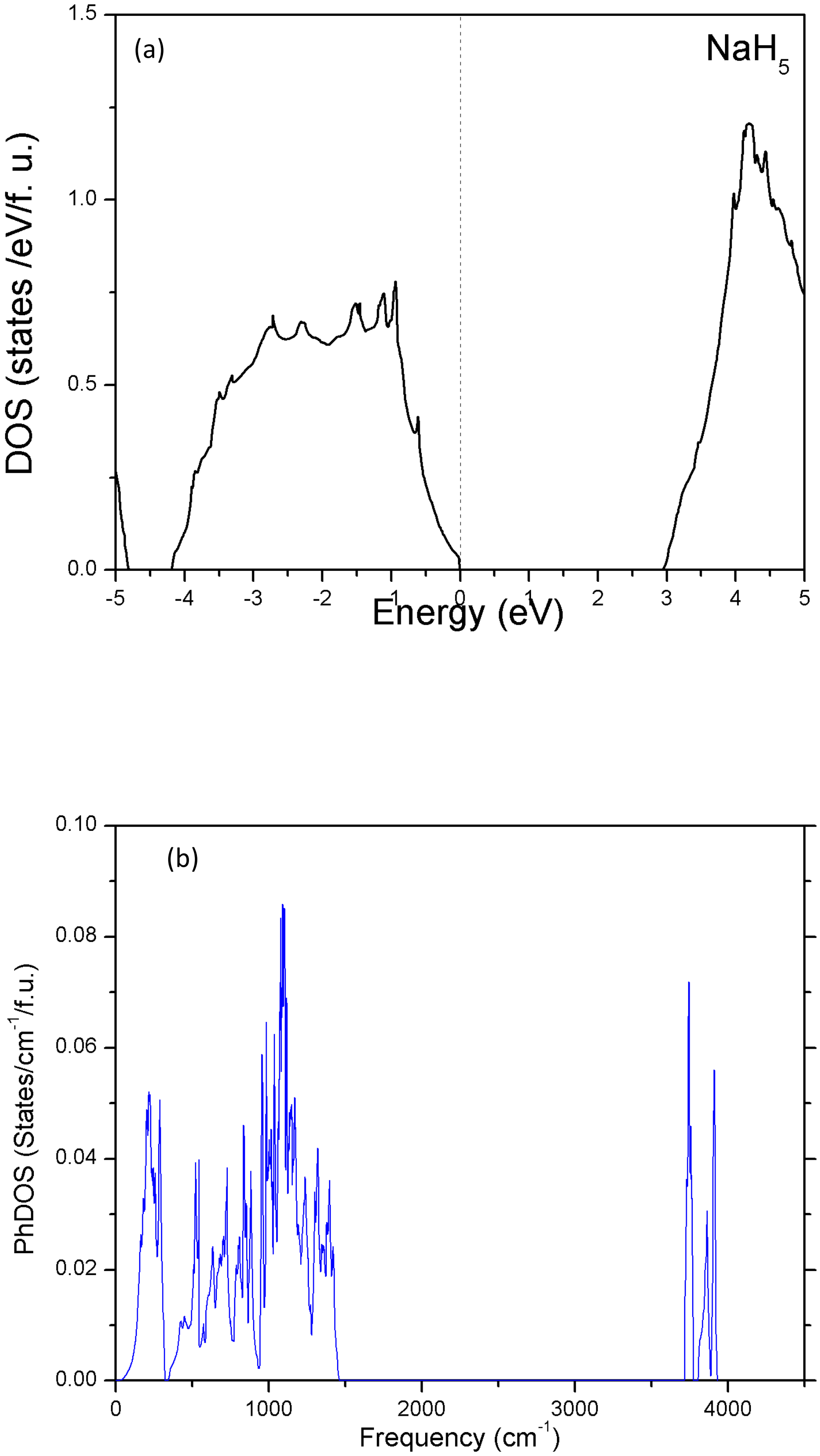

Supplementary Fig. 17. Electron and phonon DOS for NaH_5.

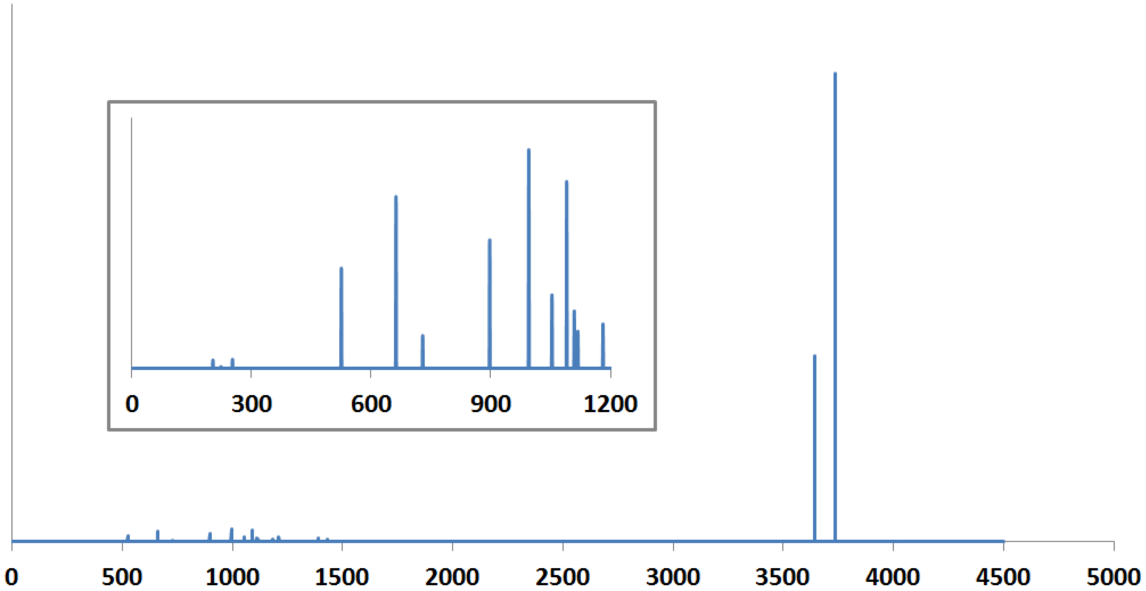

Supplementary Fig.18. Raman intensity calculated for NaH_5.

Horizontal axis in units of cm^{-1}.

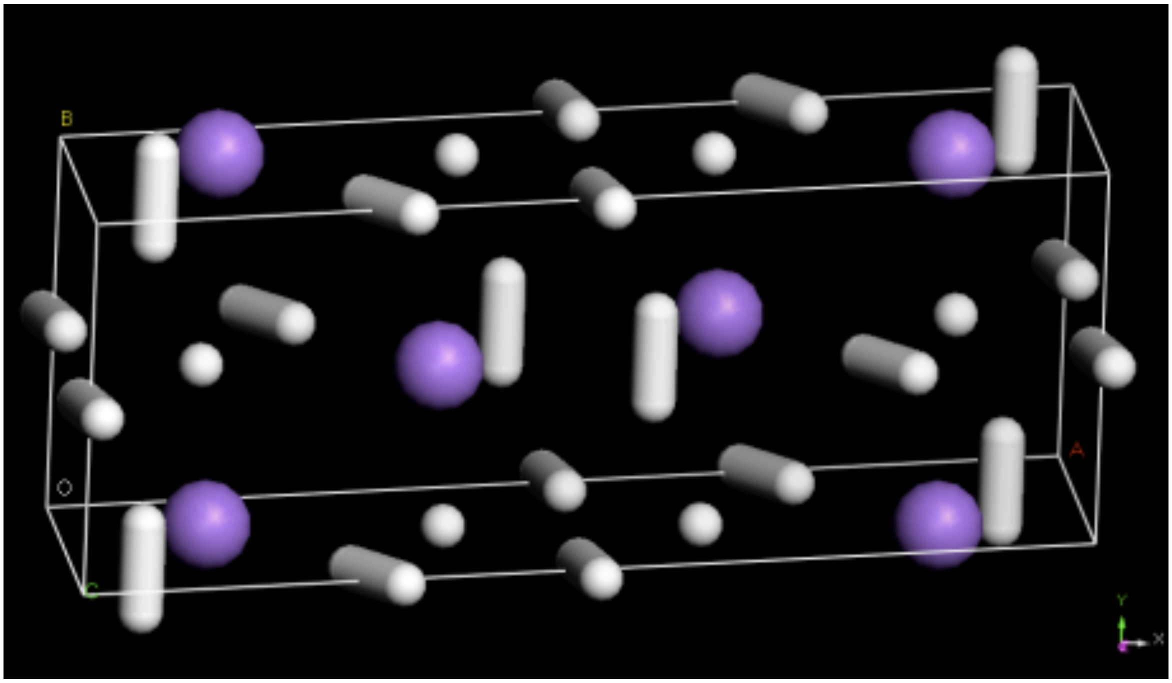

Supplementary Fig. 19. Structural unit of NaH_6.

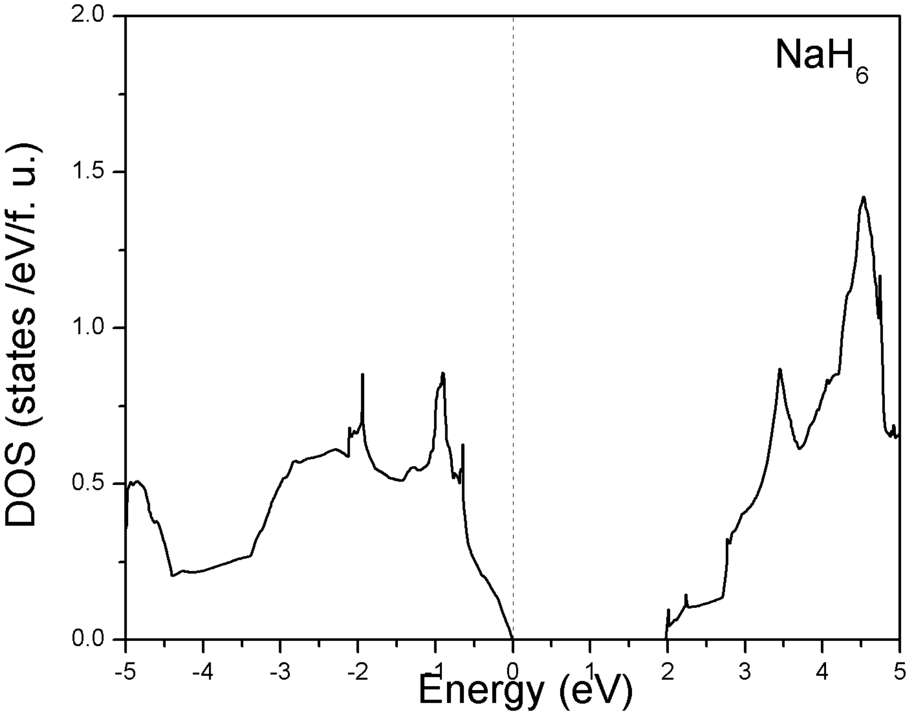

Supplementary Fig. 20. Electron DOS for NaH_6.

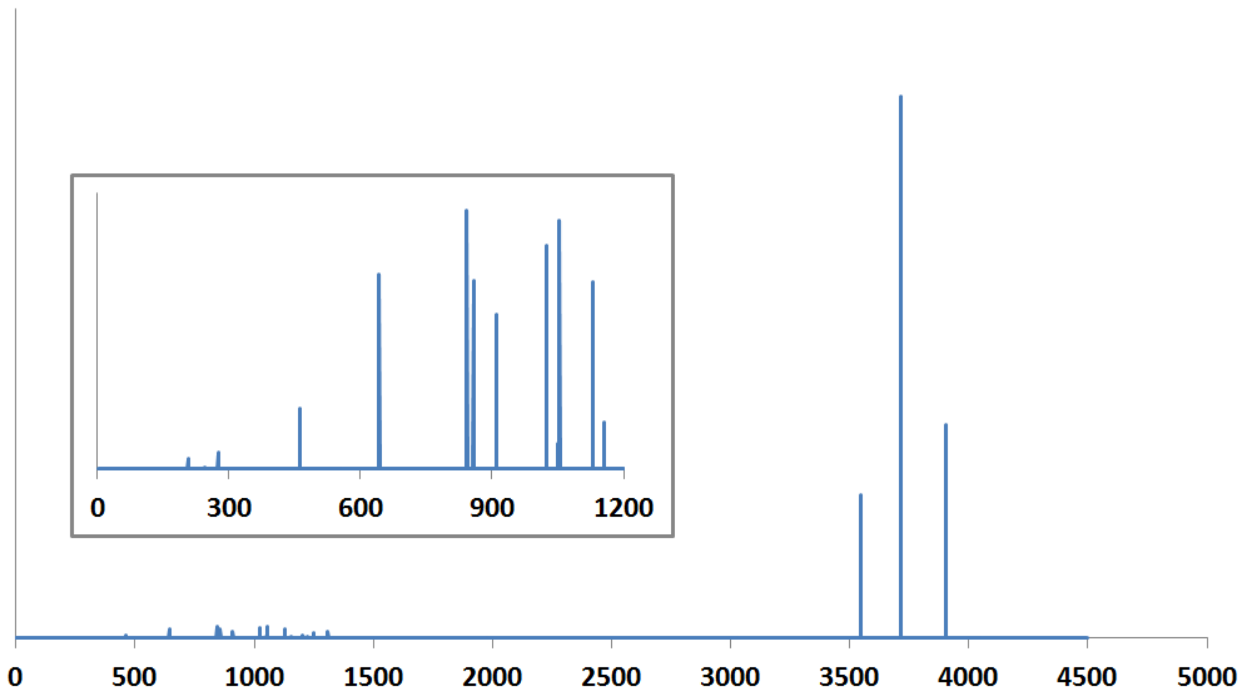

Supplementary Fig.21. Raman intensities calculated for NaH_6.

Horizontal axis in units of cm^{-1}.

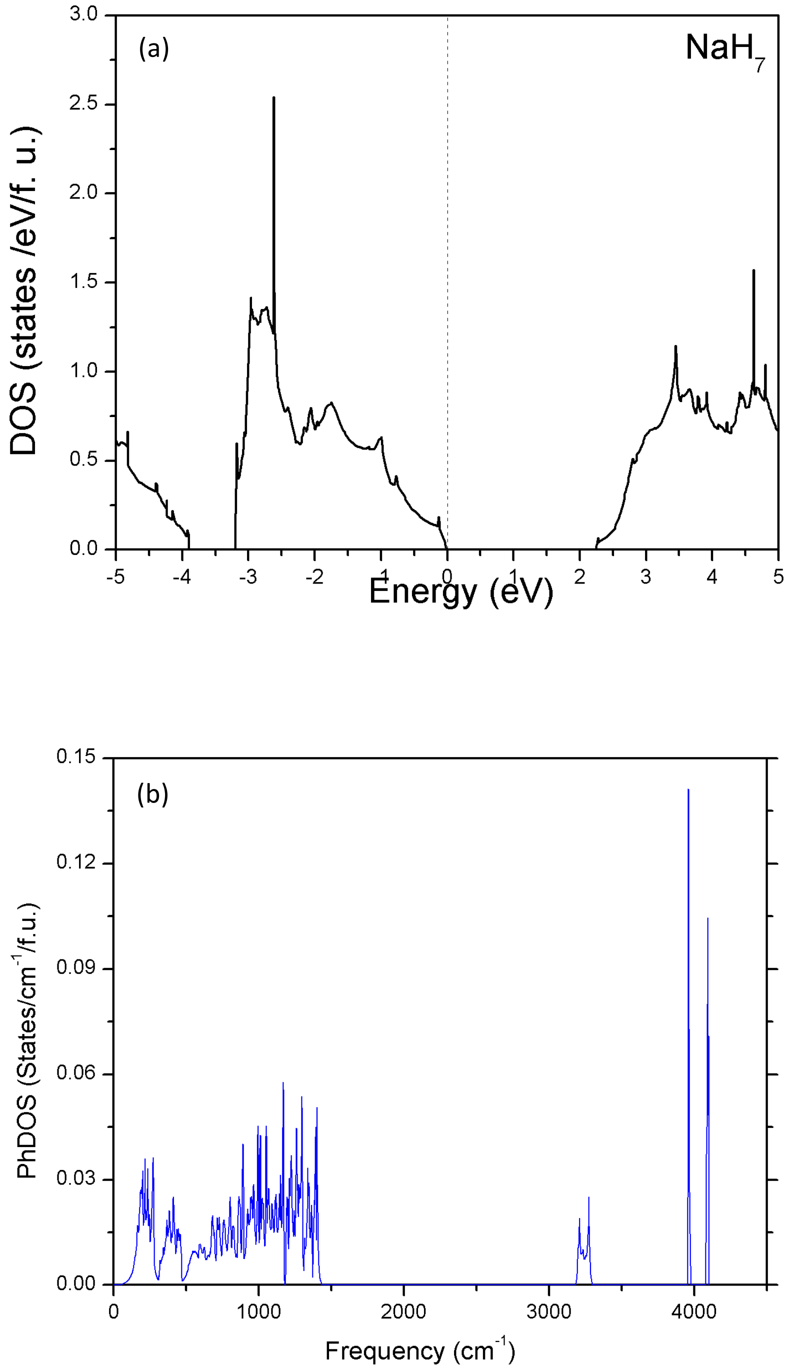

Supplementary Fig. 22. Electron (a) and phonon (b) DOS calculated for NaH_7.

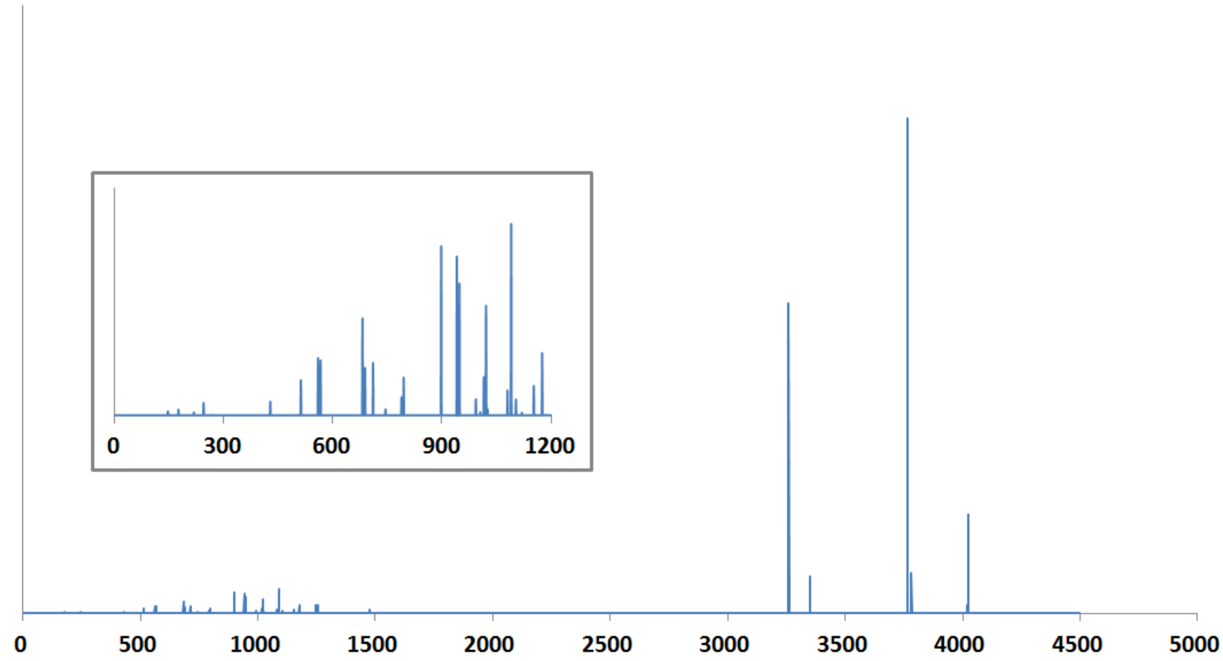

Supplementary Fig. 23. Raman intensities calculated for NaH_7.

Horizontal axis in units of cm^{-1}.

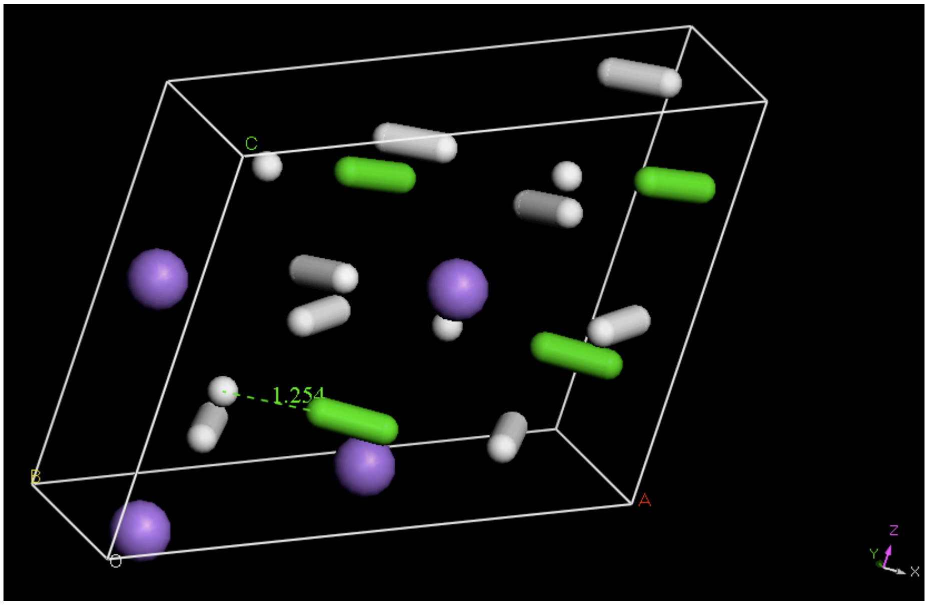

Supplementary Figure 24. Structural unit of NaH_7.

The green colored H_2 shows an extended hydrogen molecules (~0.81 Å) and its distance to the third hydrogen is 1.25 A and the corresponding angle is 176.68°.

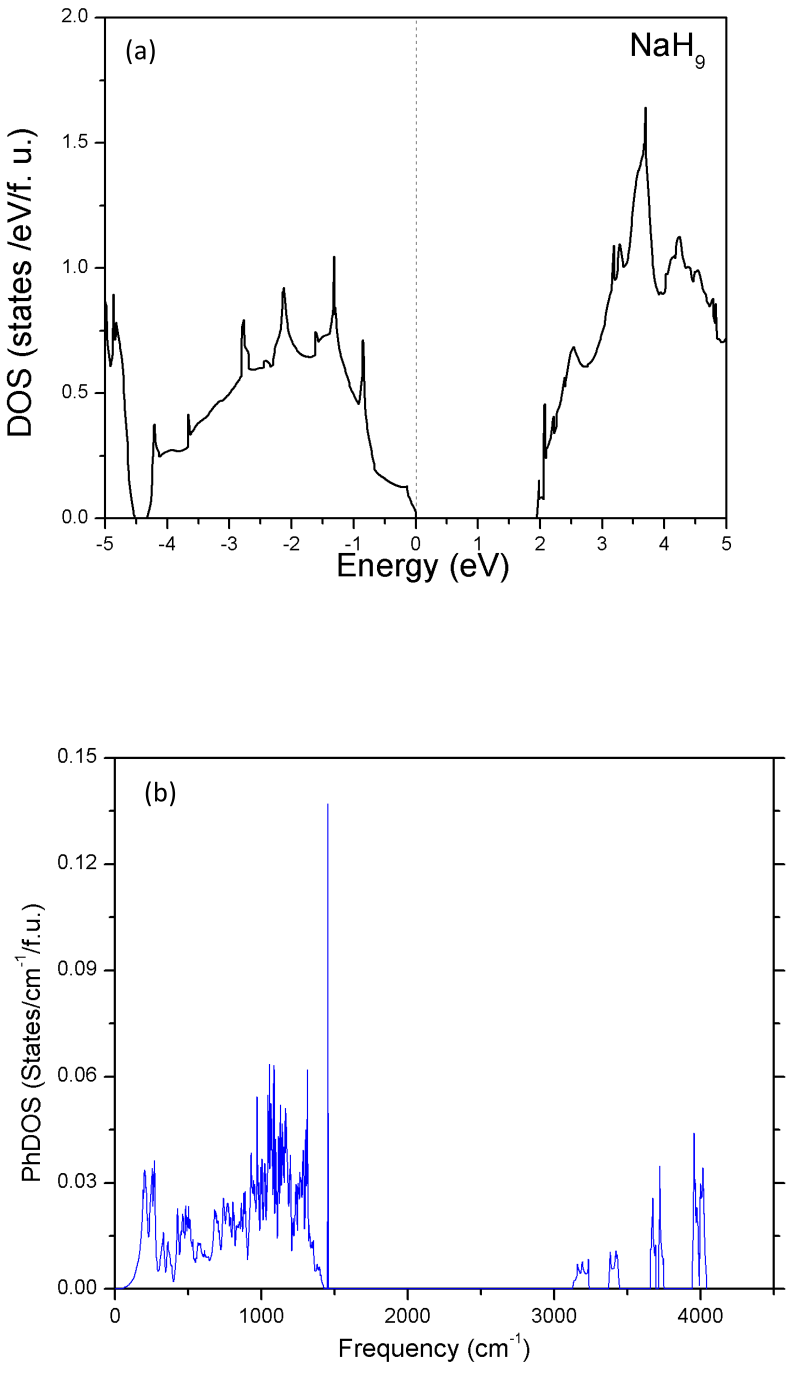

Supplementary Fig. 25. Electron (a) and phonon (b) DOS calculated for NaH_9.

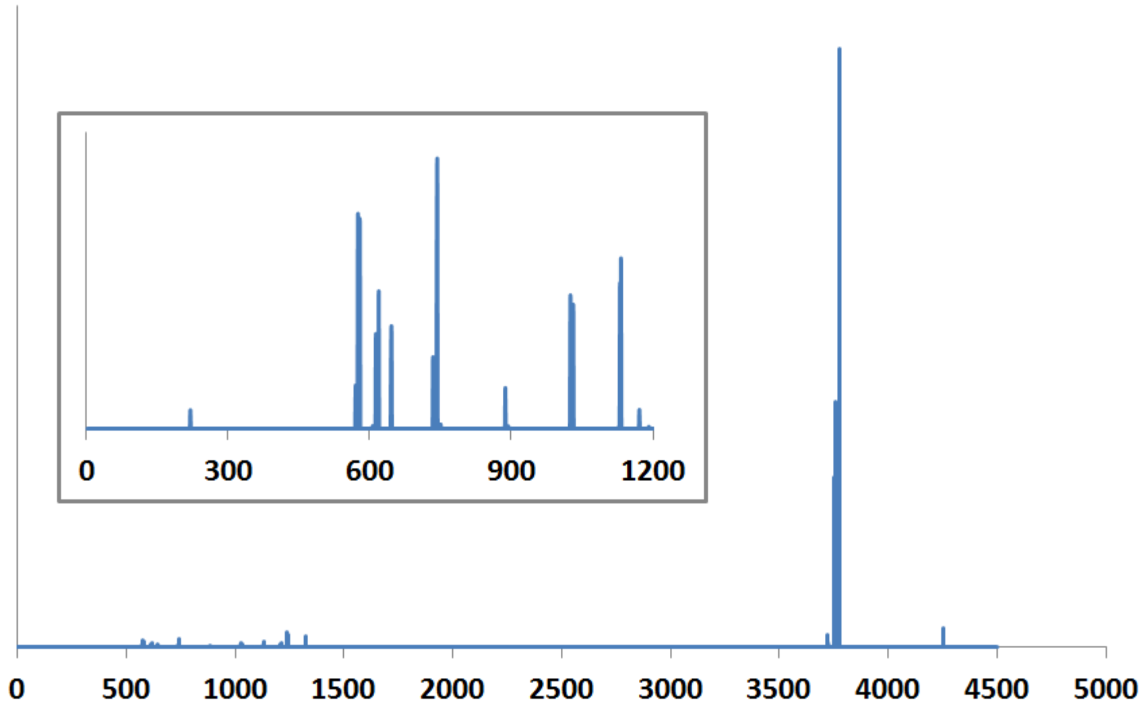

Supplementary Fig. 26. Raman intensity calculated for NaH_9.

Horizontal axis in units of cm^{-1}.

9. NaH_{11}

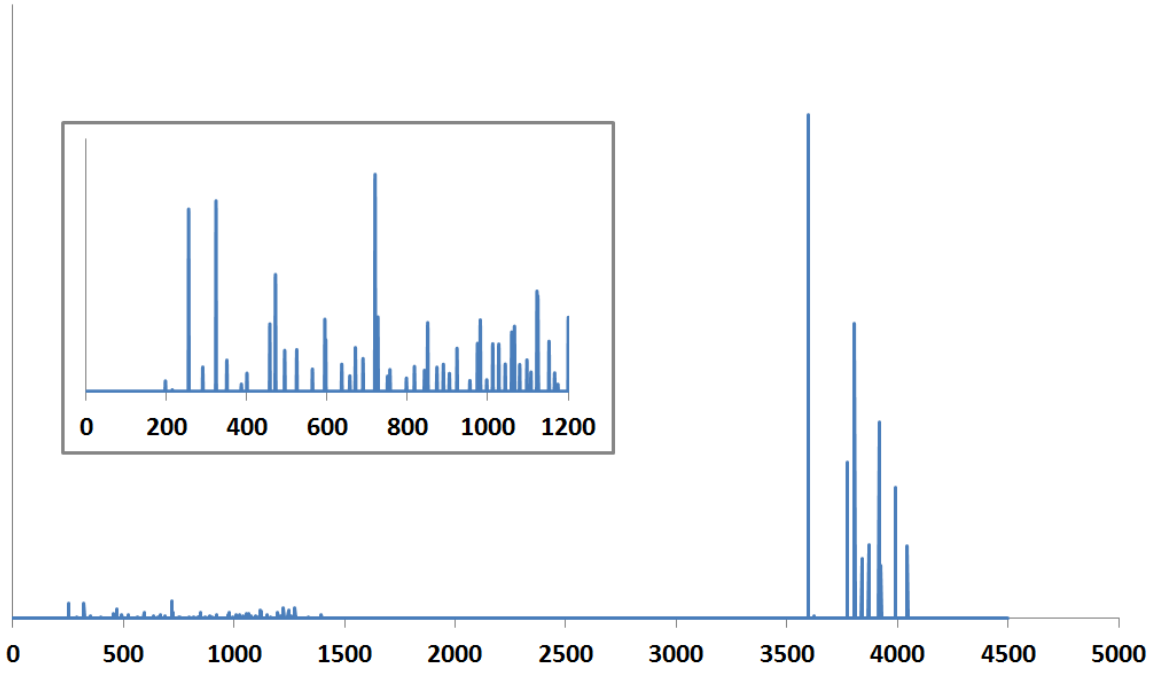

Supplementary Fig. 27. Raman intensity calculated for NaH_{11}.

Horizontal axis in units of cm^{-1}.

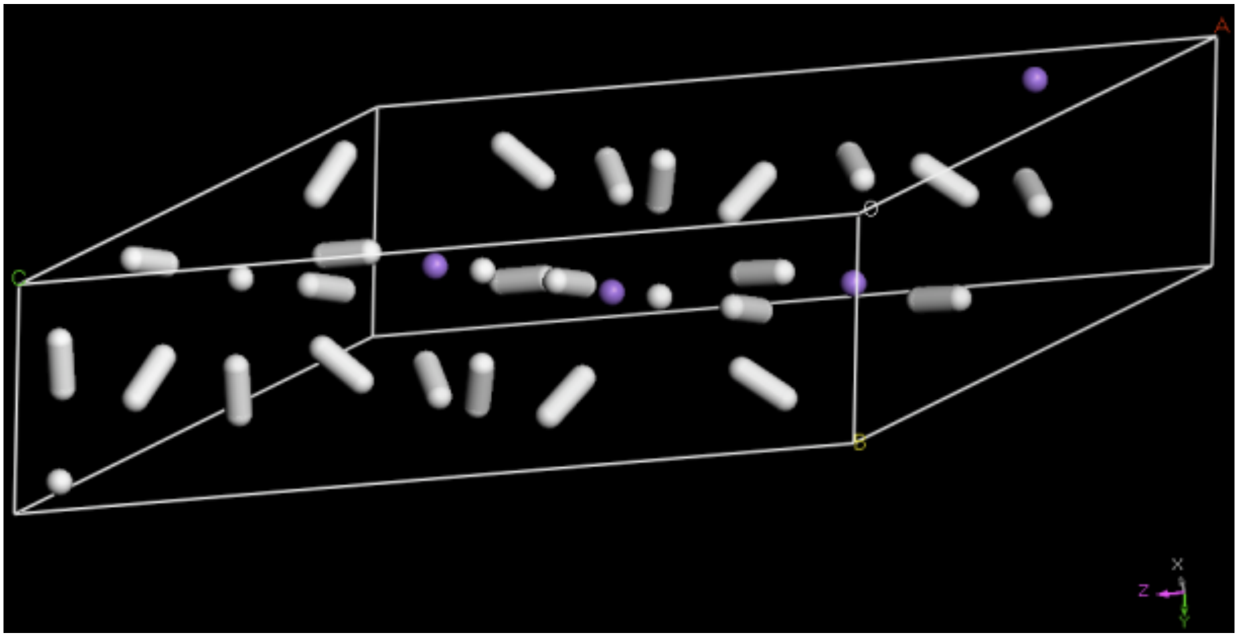

Supplementary Fig. 28. Structural unit of NaH_{13}.

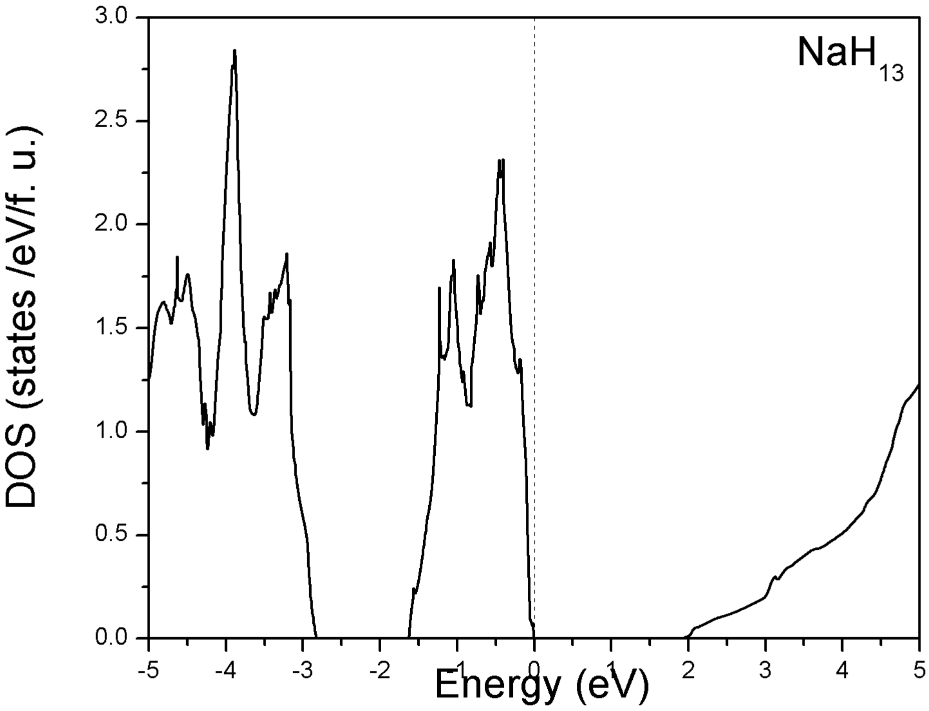

Supplementary Fig. 29. Electronic density of states for NaH_{13}.

11. Convex hull including zero point energy

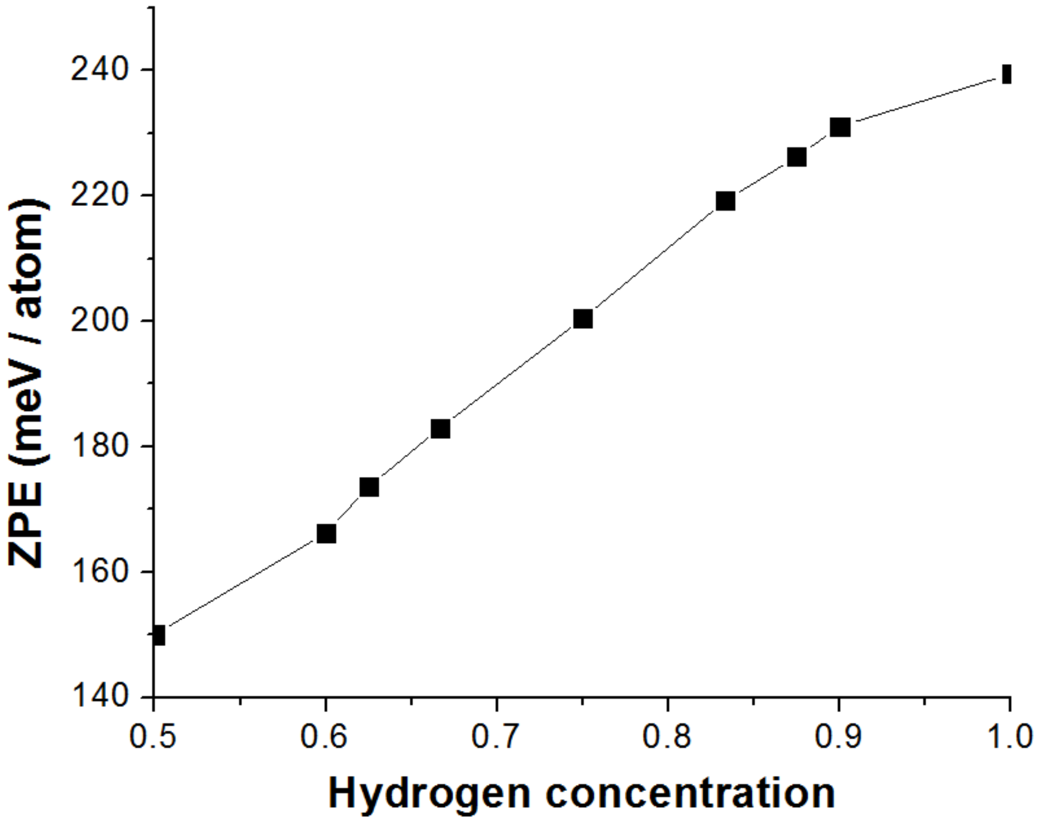

Supplementary Fig. 30. Zero point energies of various hydrides at 50 GPa are computed using calculated phonon density of states.

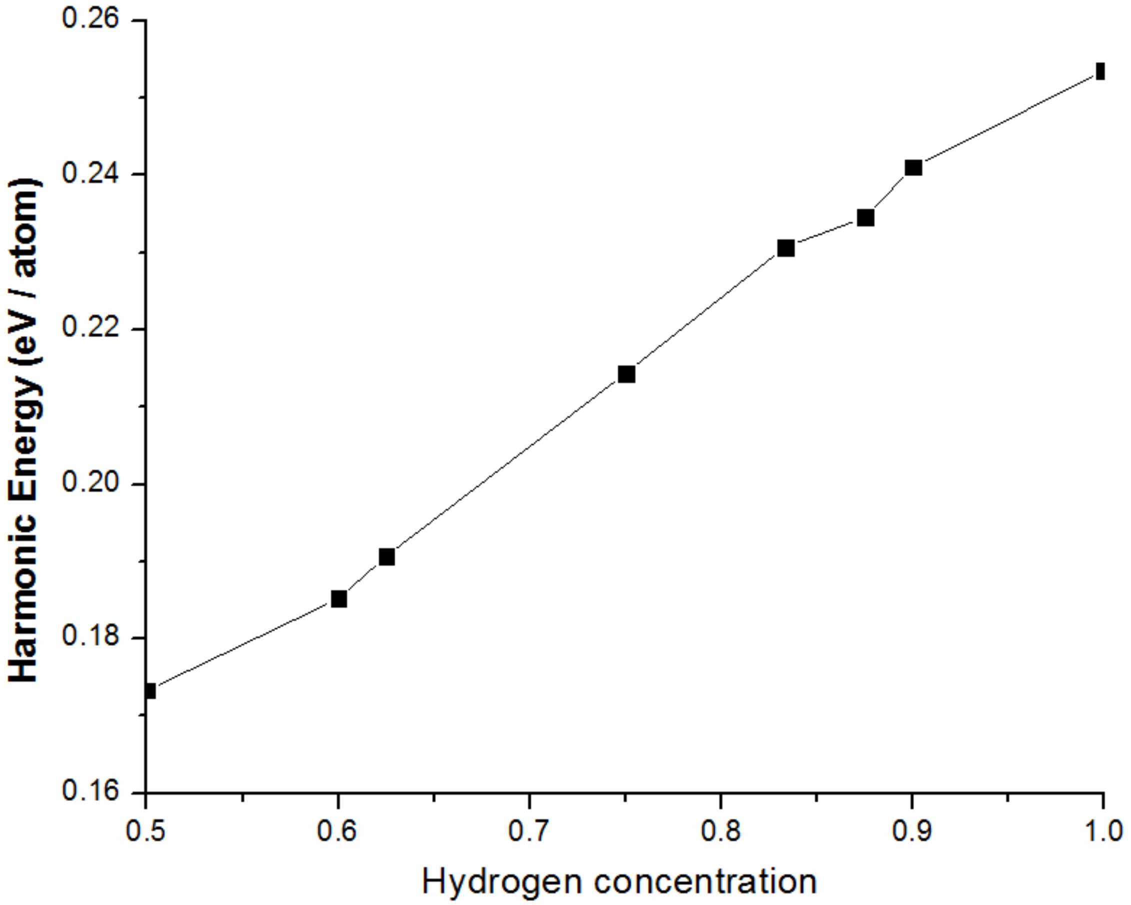

Supplementary Fig. 31 Harmonic phonon energy of various hydrides at 50 GPa and 300 K.

Supplementary Methods

Theoretically, we propose NaH_n phases stabilized at 50 GPa. Na_2H_3, Na_3H_5, NaH_2, NaH_3, NaH_5, and NaH_{13} are predicted in this work, and NaH_7 and NaH_9 were predicted in the Ref.[6] Additionally, we found a more stable NaH_6 than in the previous literature Ref. [6]; the description is given below.

Compounds

1. Na_2H_3 (4(NaH)(H_2))

Space group name P -1
Space group number 2
Setting number 1

Lattice parameters

a (Å)	b (Å)	c (Å)	alpha(°)	beta (°)	gamma (°)
5.261	5.247	5.338	68.351	67.828	68.758

Unit-cell volume = 122.522 Å^3

Structure parameters

	x	y	z
H	0.00291	0.42643	0.55028
H	0.41276	0.29901	0.58705
H	0.32849	0.82972	-0.16434
H	0.19694	0.41396	0.08798
H	0.07829	0.90023	0.31007
H	0.52885	1.03642	0.02916
Na	-0.09699	0.80851	0.07983
Na	0.30663	1.08838	0.40306
Na	0.82630	0.33276	0.32848
Na	0.40971	0.58822	0.19836

2. Na_3H_5 (3(NaH)(H_2))

Lattice type P
Space group name P -1
Space group number 2
Setting number 1

Lattice parameters

a (Å)	b (Å)	c (Å)	alpha(°)	beta (°)	gamma (°)
4.036	3.380	7.110	78.438	97.466	89.770

Unit-cell volume = 94.151 $Å^3$

Structure parameters

	x	y	z
H	0.34193	0.72082	0.57070
H	0.98195	1.04359	0.94520
H	0.94212	0.84280	0.28026
H	0.69989	0.59728	0.85613
H	0.44185	1.07686	1.01213
Na	0.15587	0.27922	0.43036
Na	0.20136	0.59407	0.86512
Na	0.55724	0.16058	0.71662

3. NaH_2 ($2(NaH)(H_2)$)

Lattice type P
Space group name P -1
Space group number 2
Setting number 1

Lattice parameters

a (Å)	b (Å)	c (Å)	alpha(°)	beta (°)	gamma (°)
3.380	5.427	4.003	71.231	90.352	105.449

Unit-cell volume = 66.755 $Å^3$

Structure parameters

	x	y	
H	0.62093	0.70196	0.67933
H	0.20490	-0.10523	0.20006
H	0.92524	0.51647	0.93117
H	1.05354	0.57672	0.46990
Na	0.61707	0.68965	0.18524
Na	0.20759	-0.10403	0.70101

4. NaH_3 ((NaH)(H_2))

Lattice type C
Space group name C m c m
Space group number 63
Setting number 1

Lattice parameters

a (Å)	b (Å)	c (Å)	alpha(°)	beta (°)	gamma (°)
3.136	6.268	3.992	90.000	90.000	90.000

Unit-cell volume = 78.475 Å^3

Structure parameters

	x	y	z
H	0.50000	0.31952	0.25000
H	0.50000	0.05278	0.95437
Na	0.00000	0.13632	0.25000

5. NaH_5 ((NaH)$_2$(H_2))

Lattice type P
Space group name P -1
Space group number 2
Setting number 1

Lattice parameters

a (Å)	b (Å)	c (Å)	alpha(°)	beta (°)	gamma (°)
3.837	3.985	3.302	90.341	92.238	86.941

Unit-cell volume = 50.373 Å^3

Structure parameters

	x	y	z
H	0.67758	0.04905	0.25100
H	0.62035	0.55914	0.17631
H	0.32786	0.37468	0.61207
H	0.14416	0.25023	0.26233
H	0.50730	0.86378	0.75771
Na	0.14403	0.74998	0.24398

6. NaH_6

Lattice type C

Space group name C 2/m

Space group number 12

Setting number 1

Lattice parameters

a (Å)	b (Å)	c (Å)	alpha(°)	beta (°)	gamma (°)
9.531	3.292	3.725	90.000	107.956	90.000

Unit-cell volume = 111.187 Å^3

Structure parameters

	x	y	z
H	-0.43325	-0.38379	0.73975
H	-0.26905	0.00000	0.15527
H	-0.62475	0.00000	0.43513
H	-0.68634	0.00000	0.06097
H	-0.50697	0.00000	0.89199
Na	-0.14575	0.00000	0.70981

It is worth to note that our predicted NaH_6 possesses a lower enthalpy than previous predicted phase [Baettig et al, 2011] by -0.034 eV / f. u.

7. NaH_{13} (NaH)6(H_2)

Lattice type C

Space group name C c

Space group number 9

Setting number 1

Lattice parameters

a (Å)	b (Å)	c (Å)	alpha(°)	beta (°)	gamma (°)
7.067	4.044	12.451	90.000	147.700	90.000

Unit-cell volume = 190.141 Å^3

Structure parameters

	x	y	z
H	-1.67361	0.58093	-1.03494
H	-1.31867	0.39520	-0.83900

H	-0.82465	0.34060	-0.83559
H	-0.10464	0.39647	-0.33602
H	0.09420	0.37775	-0.23598
H	0.30058	0.58507	-0.23577
H	-1.17380	0.67468	-1.03633
H	0.00653	0.52932	-0.55087
H	-1.28720	0.17954	-1.05079
H	-0.49141	0.61717	-0.54889
H	-1.26603	0.58290	-1.03597
H	-1.46666	0.78447	-1.03503
H	0.29026	0.17487	-0.24110
Na	-0.01472	0.88567	-0.28996
